# Correction: A Mixture of Delta-Rules Approximation to Bayesian Inference in Change-Point Problems

**DOI:** 10.1371/journal.pcbi.1006210

**Published:** 2018-06-26

**Authors:** Robert C. Wilson, Matthew R. Nassar, Gaia Tavoni, Joshua I. Gold

**Affiliations:** 1 Department of Psychology and Cognitive Science Program, University of Arizona, Tucson, Arizona, United States of America; 2 Department of Psychology, Brown University, Providence, Rhode Island, United States of America; 3 Department of Neuroscience, University of Pennsylvania, Philadelphia, Pennsylvania, United States of America

This Research Article reports that human behavior is best fit by a mixture-of-delta-rules model with a small number of nodes. The authors noticed that there are several errors in this paper. Most of the errors are typos that do not reflect how they implemented the algorithm in code. One issue is more serious and changes some of the results in Figs 8 and 9. In addition this more serious issue leads to minor changes in Figs 1, 5, 6, 10, 11 and S1 and Tables 1 and S1.

**Fig 1:**
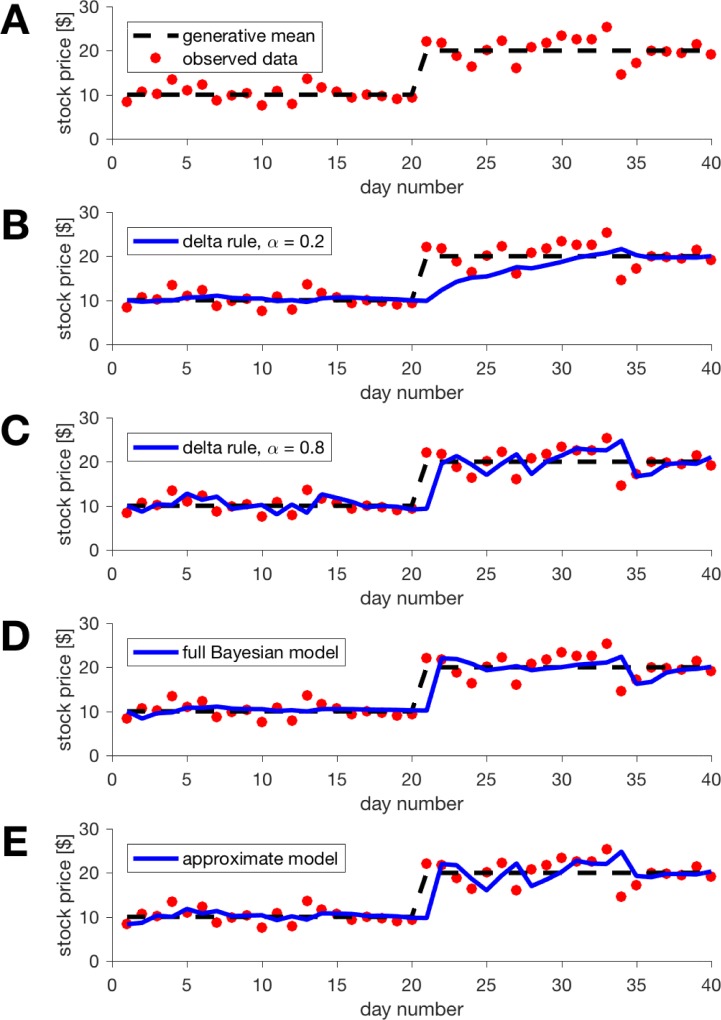
An example change-point problem with a single change-point at time 20 (A) and an illustration of the performance of di_erent algorithms at making predictions(B-E). (B) The Delta rule model with learning rate parameter _ = 0:2 performs well before the change-point but poorly immediately afterwards. (C) The Delta rule model with learning rate _ = 0:8 responds quickly to the change-point but has noisier estimates overall. (D) The full Bayesian model dynamically adapts its learning rate to minimize error overall. (E) Our approximate model shows similar performance to the Bayesian model but is implemented at a fraction of the computational cost and in a biologically plausible manner.

**Table 1:** Table of mean fit parameter values for all models ± s.e.m.

Model	Hazard rate, *h*	Decision noise, *σ*_*d*_	Learning rate(s), *α*
Full	0.50 ± 0.04	13.39 ± 0.52	
Nassar et al.	0.45 ± 0.04	8.35 ± 0.87	
1 node		8.7 ± 0.72	0.88 ± 0.014
2 nodes	0.36 ± 0.04	7.41 ± 0.67	0.92 ± 0.01 0.43 ± 0.03
3 nodes	0.44 ± 0.04	7.8 ± 0.76	0.91 ± 0.01 0.46 ± 0.02 0.33 ± 0.02

None of the errors change the major conclusions of the paper: i.e. that human behavior is best fit by a mixture-of-delta-rules model with a small number of nodes. Here the authors outline the errors in detail and the changes they have made to the manuscript to address them. In addition, the authors have the shared code implement algorithm in the updated paper on GitHub: github.com/bobUA/2013WilsonEtAlPLoSCB.

## Major issue with change-point prior in Fig 3

The most serious error arises in Fig 3 where the implied change-point prior for the reduced model does not match the change-point prior derived in Eqs 26–31. In particular the edge weights in Fig 3 should be changed to reflect the true change-point prior.

**Fig 3:**
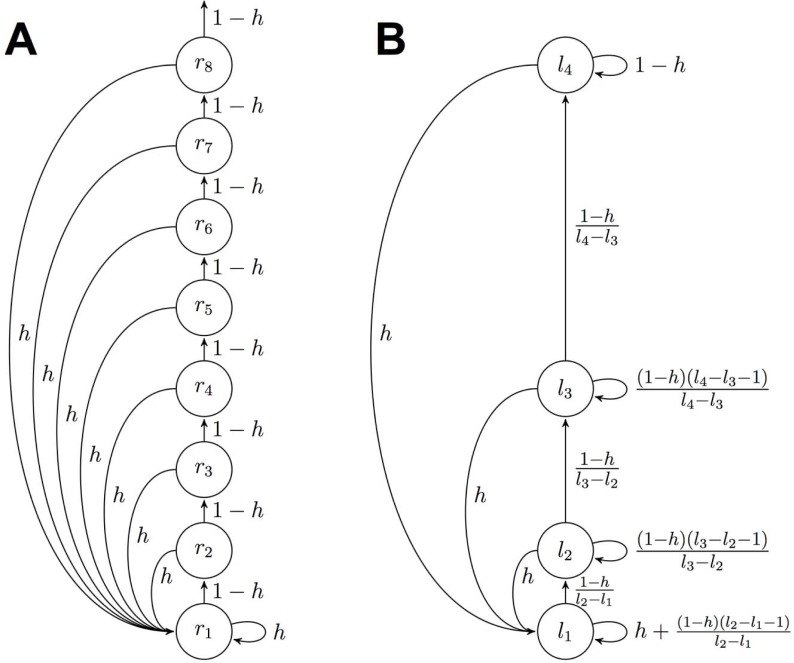
Schematic of the message passing algorithm for the full (A) and approximate (B) algorithms. For the approximate algorithm we only show the case for li+1 li + 1.

**Fig 5:**
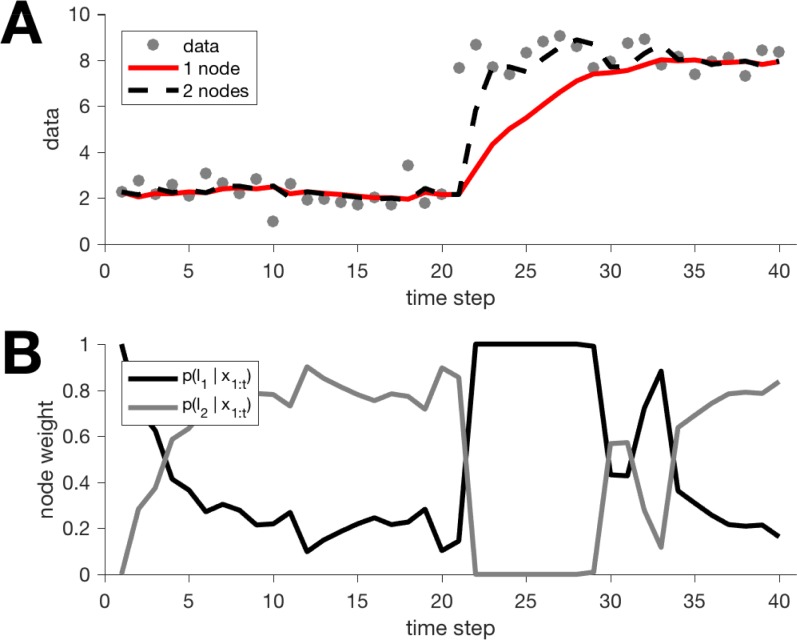
Output of one- and two- node models on a simple change-point task. (A) Predictions from the one- and two-node models. (B) Evolution of the node weights for the two-node model.

**Fig 6:**
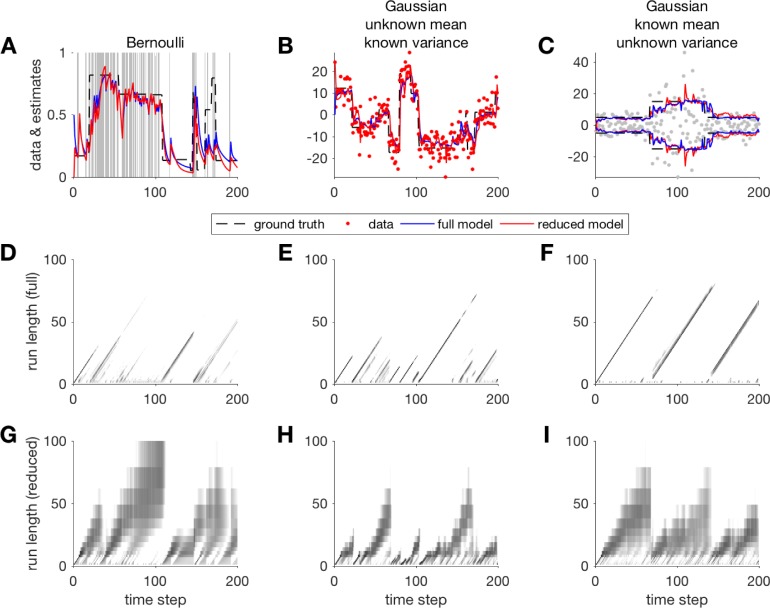
Examples comparing estimates and run-length distributions from the full Bayesian model and our reduced approximation for the cases of Bernoulli data (A, D, G), Gaussian data with unknown mean (B, E, H), and Gaussian data with a constant mean but unknown variance (C, F, I). (A, B, C) input data (grey), model estimates (blue: full model; red: reduced model), and the ground truth generative parameter (mean for A and B, standard deviation in C; dashed black line). Run-length distributions computed for the full model (D, E, F) and reduced model (G, H, I) are shown for each of the examples.

**Fig 8:**
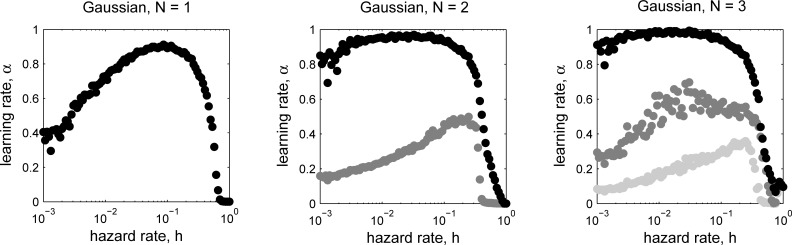
Optimal learning rates, corresponding to the lowest relative error (see _gure 7), as a function of hazard rate and number of nodes. Gaussian case with 1 (left), 2 (center), or 3 (right) nodes.

**Fig 9:**
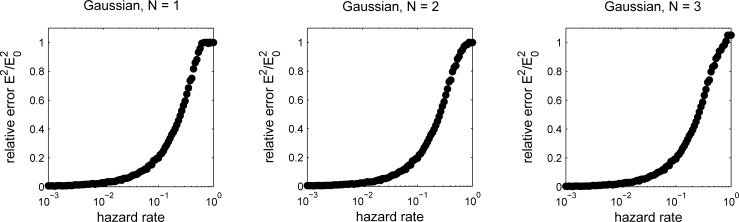
Error (normalized by the variance of the prior, E2 0) computed from simulations as a function of hazard rate for the reduced model at the optimal parameter settings as shown in _gure 8. Gaussian case with 1 (left), 2 (center), or 3 (right) nodes.

**Fig 10:**
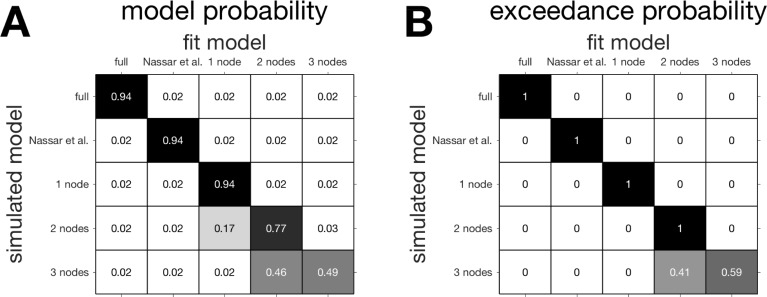
Confusion matrices. (A) The confusion matrix of model probability, the estimated fraction of data simulated according to one model that is _t to each of the models. (B) The confusion matrix of exceedance probability, the estimated probability at the group level that a given model has generated all the data.

**Fig 11:**
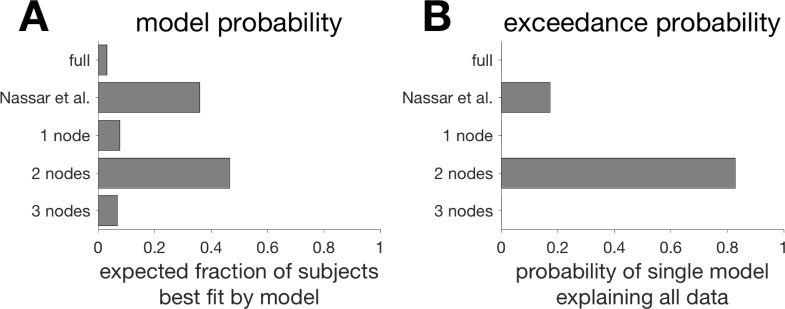
Results of the model-_tting procedure using the method of [28]. (A) The model probability for each of the _ve models. This measure reports the estimated probability that a given subject will be best _t by each of the models. (B) The exceedance probability for each of the _ve models. This measure reports the probability that each of the models best explains the data from all subjects.

Unfortunately, this error in Fig 3 was carried over into the code and we actually used this (incorrect) change-point prior in our simulations. Fortunately, however, updating the code to include the new change-point prior has a relatively small effect on most results. Thus there are only minor, quantitative changes required in Figs 1, 5 and 6. Likewise the model fitting results (Figs 10, 11 and S1 and Tables 1 and S1) are slightly changed, with the biggest change being that the 2-node, not the 3-node, model now best fits human behavior.

The one place our use of the incorrect change-point prior has a large effect is in the section “Performance of the reduced model relative to ground truth.” In particular, when we use the correct change-point prior, the simplifying assumption in Eq 48 no longer holds. That is, we have

$$\langle p_{i}\;p_{j}\;\mu_{i}\;\mu_{j}\rangle \neq \langle p_{i}\rangle \langle p_{j}\rangle \langle \mu_{i}\mu_{j}\rangle$$

This invalidates the analysis of the two- and three-node cases in Figs 8 and 9. Specifically, the results in Fig 8B, 8C, 8E and 8F and the results in Fig 9B, 9C, 9E and 9F no longer hold.

We have therefore instead computed numerical solutions to the optimization problem for the Gaussian case. As shown in the updated figures, the results are quantitatively different from the original paper, as to be expected given the problems discussed above, but are qualitatively consistent with our previous findings. Thus, the new results do not change the main conclusions, most importantly that performance of the algorithm improves substantially from 1 to 2 nodes, but only incrementally from 2 to 3 nodes.

## Typos

In addition to the major error with the change-point prior, the original paper also contains a number of typos. These do not reflect how the algorithm was actually implemented. We now list these changes in detail:

In Eqs 6 and 7, the sums should start from *r*_*t*+1_ = 0 not *r*_*t*+1_ = 1; i.e. they should read

$$p(x_{t+1}|x_{1:t})=\sum_{r_{t+1=0}}^{t+1}p(x_{t+1}|r_{t+1})p(r_{t+1}|x_{1:t})$$

and

$$p(r_{t}|x_{1:t})\propto p(x_{t}|r_{t})p(r_{t}|x_{1:t-1})$$

$$=p(x_{t}|r_{t})\sum_{r_{t-1}=0}^{t-1}p(r_{t}|r_{t-1})p(r_{t-1}|x_{1:t-1})$$

Eq 14 has sign error and should read

$$\tilde{A}(v_{p},\chi_{p})=\log\left(\frac{\sigma }{\sqrt{v_{p}}}\right)+\frac{\chi_{p}^{2}}{2\sigma^{2}v_{p}}$$

Eq 15 should read

$$p(\eta |r_{t})=p(\eta |v_{t}^{r_{t}},\chi_{t}^{r_{t}})$$

$$=\tilde{H}(\eta )\exp\left(\eta^{T}\chi_{t}^{r_{t}}-v_{t}^{r_{t}}A(\eta )-\tilde{A}(v_{t}^{r_{t}},\chi_{t}^{r_{t}})\right)$$

where the only change is that the order of arguments into $\tilde{A}$ have been switched.

Eq 32 for the weight of the increasing node should read

$$\begin{array}{c} \frac{1-h}{l_{j+1}-l_{j}}p(l_{j}|x_{1:t})\;\text{for}\;l_{j+1}>l_{j}+1\\ (1-h)p(l_{j}|x_{1:t})\;\text{otherwise} \end{array}$$

Eq 33 for the weight of the self node should read

$$\begin{array}{c} \frac{l_{j+1}-l_{j}-1}{l_{j+1}-l_{j}}(1-h)p(l_{j}|x_{1:t})\;\text{for}\;l_{j+1}>l_{j}+1\\ 0\;\text{otherwise} \end{array}$$

Eq 34 should read

$$hp(l_{j}|x_{1:t})$$

Eq 38 should read

$$p(l_{1}|x_{1:t})\propto p(x_{t}|l_{1})(p(l_{1}|l_{1})p(l_{1}|x_{1:t-1})+p(l_{1}|l_{2})p(l_{2}|x_{1:t-1}))$$

$$=p(x_{t}|l_{1})\left((h+\frac{\left(1-h)(l_{2}-l_{1}-1\right)}{l_{2}-l_{1}})p(l_{1}|x_{1:t-1})+hp(l_{2}|x_{1:t-1})\right)$$

$$=p(x_{t}|l_{1})\left(h+\frac{\left(1-h)(l_{2}-l_{1}-1\right)}{l_{2}-l_{1}}p(l_{1}|x_{1:t-1})\right)$$

Eq 39 should read …

$$p(l_{2}|x_{1:t})\propto p(x_{t}|l_{2})(p(l_{2}|l_{1})p(l_{1}|x_{1:t-1})+p(l_{2}|l_{2})p(l_{2}|x_{1:t-1}))=p(x_{t}|l_{2})\left(\frac{1-h}{l_{2}-l_{1}}p(l_{1}|x_{1:t-1})+(1-h)p(l_{2}|x_{1:t-1})\right)$$

$$=p(x_{t}|l_{2})(1-h)\left(1-\frac{l_{2}-l_{1}-1}{l_{2}-l_{1}}\right)p(l_{1}|x_{1:t-1})$$

Eq 40 should read

$$p(x_{t}|l_{i})=\frac{1}{\sigma \sqrt{2\pi }}\sqrt{\frac{l_{i}+v_{p}}{1+l_{i}+v_{p}}}\exp\left(-\frac{1}{2\sigma^{2}}(\frac{l_{i}+v_{p}}{{1+l}_{i}+v_{p}}){\left(x_{t}-\mu_{t}^{l_{i}}\right)}^{2}\right)$$

Eq 41 should read …

$$\eta =\log\left(\frac{\mu }{1-\mu }\right);\;H(x)=1;\;U(x)=x;\;A(\eta )=-\log(1-\mu )=\log\left(1+\exp\eta \right)$$

## Supporting information

S1 Fig Histograms of fit parameter values for all models. Each column represents a model, with the name of the model given at the top. Each row represents a single variable going, in order from top to bottom: hazard rate, decision noise standard deviation, learning rate 1, learning rate 2 and learning rate 3. Where a particular model does not have a particular parameter that box is left empty.
(EPS)

S1 Table Table showing correlation coefficient between simulated and fit parameter values. (PDF)

S1 Text Derivation of error relative ground truth. (PDF)

S2 Text Corrected manuscript. (PDF)
