# Supplementary figures and images for: A Mixture of Delta-Rules Approximation to Bayesian Inference in Change-Point Problems
Source: PLoS Comput Biol. 2013 Jul 25;9(7):e1003150. doi: 10.1371/journal.pcbi.1003150 (PMC3723502; doi:10.1371/journal.pcbi.1003150)

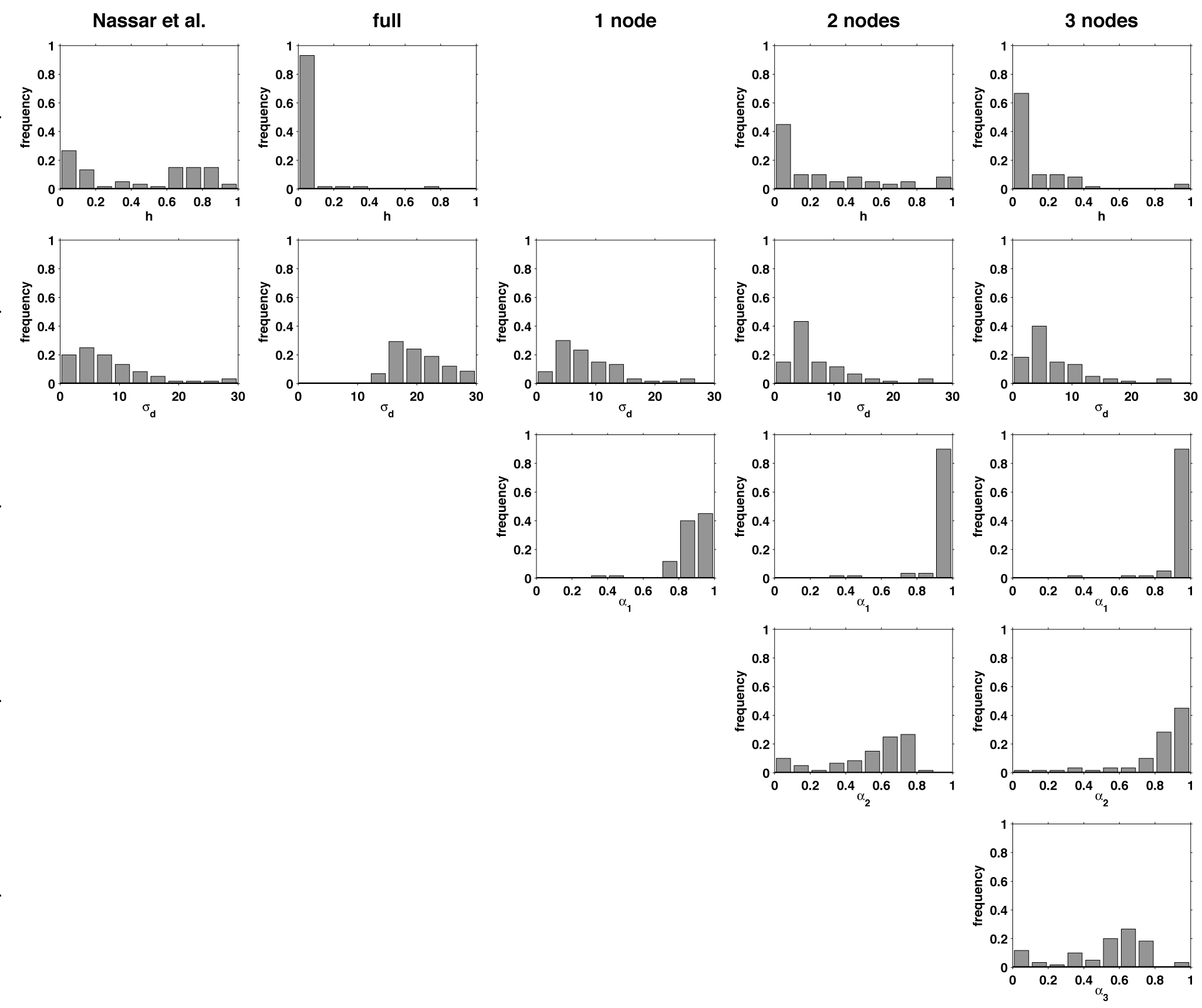

Supplement: Figure S1 — Histograms of fit parameter values for all models. Each column represents a model, with the name of the model given at the top. Each row represents a single variable going, in order from top to bottom: hazard rate, decision noise standard deviation, learning rate 1, learning rate 2 and learning rate 3. Where a particular model does not have a particular parameter that box is left empty. (TIF) [file pcbi.1003150.s001.tif]
