# Supplementary material for: A Mixture of Delta-Rules Approximation to Bayesian Inference in Change-Point Problems
Source: PLoS Comput Biol. 2013 Jul 25;9(7):e1003150. doi: 10.1371/journal.pcbi.1003150 (PMC3723502; doi:10.1371/journal.pcbi.1003150)
Supplement: Table S1 — Table showing correlation coefficient between simulated and fit parameter values. (PDF) [file pcbi.1003150.s002.pdf]

# Tables

| Model | parameter | correlation coefficient | $p$-value |
|---|---|---|---|
| Nassar et al. | $h$ | $r = 0.46$ | $p = 0.0002282$ |
| | $\sigma_d$ | $r = 0.61$ | $p =$ 2.0938e-07 |
| Full | $h$ | $r = 0.36$ | $p = 0.0071286$ |
| | $\sigma_d$ | $r = 0.51$ | $p =$ 6.8173e-05 |
| 1-node | $\sigma_d$ | $r = 0.59$ | $p =$ 6.2369e-07 |
| | $\alpha_1$ | $r = 0.56$ | $p =$ 3.0972e-06 |
| 2-node | $h$ | $r = 0.49$ | $p =$ 6.5561e-05 |
| | $\sigma_d$ | $r = 0.55$ | $p =$ 4.9789e-06 |
| | $\alpha_1$ | $r = 0.53$ | $p =$ 1.4408e-05 |
| | $\alpha_2$ | $r = 0.56$ | $p =$ 3.5277e-06 |
| 3-node | $h$ | $r = 0.44$ | $p = 0.00036849$ |
| | $\sigma_d$ | $r = 0.55$ | $p =$ 5.9393e-06 |
| | $\alpha_1$ | $r = 0.27$ | $p = 0.036072$ |
| | $\alpha_2$ | $r = 0.42$ | $p = 0.0007209$ |
| | $\alpha_3$ | $r = 0.63$ | $p =$ 8.3748e-08 |

**Figure S 1.** Table showing correlation coefficient between simulated and fit parameter values.
