# Supplementary material for: A Mixture of Delta-Rules Approximation to Bayesian Inference in Change-Point Problems
Source: PLoS Comput Biol. 2013 Jul 25;9(7):e1003150. doi: 10.1371/journal.pcbi.1003150 (PMC3723502; doi:10.1371/journal.pcbi.1003150)
Supplement: Text S1 — Derivation of error relative ground truth. (PDF) [file pcbi.1003150.s003.pdf]

Supplementary Material: A mixture of Delta-rules approximation to Bayesian inference in change-point problems

Robert C. Wilson[1,*], Matthew R. Nassar[2], Joshua I. Gold[2]
1 Princeton Neuroscience Institute, Princeton University, Princeton, NJ 08540
2 Department of Neuroscience, University of Pennsylvania, Philadelphia, PA 19104
∗ E-mail: rcw2@princeton.edu

Full derivation of approximate error vs number of nodes

In order to compute the mean squared error we need expressions for four terms in equation 47 of the main text. These are the three terms related to the mean; i.e. $\left\langle \left(m^G\right)^2 \right\rangle$, $\left\langle \mu_i m^G \right\rangle$, and $\langle \mu_i \mu_j \rangle$, and the average run-length distribution $\langle p_i \rangle$. We now derive these terms one at a time.

Term 1: $\left\langle \left(m^G\right)^2 \right\rangle$

The simplest of these is just the square mean of the prior distribution over m^G the ground truth mean; i.e.,

$$\left\langle \left(m^G\right)^2 \right\rangle = \int \left(m^G\right)^2 p(m^G|v_p, \chi_p) dm^G \tag{1}$$

This term is defined by our choice of the prior.

Term 2: $\left\langle \mu_i m^G \right\rangle$

To compute the second term, $\left\langle \mu_i m^G \right\rangle$, we first express the means, μ_i, of the individual Delta rules as weighted sum of all previous data points; i.e.,

$$\begin{aligned} \mu_i &= \sum_{a=1}^{t} \alpha_i (1-\alpha_i)^{t-a} x_a \\ &= \sum_{a=1}^{t} \kappa_{ia} x_a \end{aligned} \tag{2}$$

where the kernel $\kappa_{ia} = \alpha_i(1-\alpha_i)^{t-a}$. Using this kernel expression for μ_i, we can write

$$\left\langle \mu_i m^G \right\rangle = \sum_{a=1}^{t} \kappa_{ia} \left\langle x_a m^G \right\rangle \tag{3}$$

If there is no change-point between time a and time $t+1$, then x_a is sampled from a distribution with mean m^G and we have

$$\left\langle x_a m^G \right\rangle_{\text{no change-point}} = \left\langle \left(m^G\right)^2 \right\rangle \tag{4}$$

which is just the square mean of the prior over m^G. Conversely, if there is a change-point between a and $t+1$, then x_a comes from a different distribution and we have

$$\left\langle x_a m^G \right\rangle_{\text{change-point}} = \left\langle m_p m^G \right\rangle = m_p \left\langle m^G \right\rangle = m_p^2 \tag{5}$$

where m_p is the mean of the prior distribution over m^G.

Finally, to compute $\langle x_a m^G \rangle$ we need to marginalize over the two possibilities that a change-point has occurred or not. The probability that there is no change-point between times a and $t+1$ is $(1-h)^{t-a+1}$ and a change happens with probability $1-(1-h)^{t-a+1}$. These probabilities give us the following expression for $\langle x_a m^G \rangle$,

$$\begin{aligned} \langle x_a m^G \rangle &= (1-h)^{t-a+1} \langle x_a m^G \rangle_{\text{no change-point}} + \left(1-(1-h)^{t-a+1}\right) \langle x_a m^G \rangle_{\text{change-point}} \\ &= (1-h)^{n+1} \left(\left\langle \left(m^G\right)^2 \right\rangle - m_p^2 \right) + m_p^2 \\ &= (1-h)^{n+1} \xi_0 + \xi_1 \end{aligned} \tag{6}$$

where we have defined $n = t-a$, $\xi_0 = \left\langle \left(m^G\right)^2 \right\rangle - m_p^2$ and $\xi_1 = m_p^2$. Thus we can write

$$\begin{aligned} \langle \mu_i m^G \rangle &= \sum_{n=0}^{t-1} \alpha_i (1-\alpha_i)^n \left(\xi_0 (1-h)^{n+1} + \xi_1 \right) \\ &= \frac{\xi_0 \alpha_i (1-h) \left(1-(1-\alpha_i)^t (1-h)^t\right)}{1-(1-\alpha_i)(1-h)} + \xi_1 \left(1-(1-\alpha_i)^t\right) \end{aligned} \tag{7}$$

Term 3: $\langle \mu_i \mu_j \rangle$

$\langle \mu_i \mu_j \rangle$, is calculated in a similar manner. Using the kernel expression for μ_i (equation 2), we can write

$$\begin{aligned} \langle \mu_i \mu_j \rangle &= \sum_{a=1}^{t} \sum_{b=1}^{t} \kappa_{ia} \kappa_{jb} \langle x_a x_b \rangle \\ &= C(0) \sum_{a=1}^{t} \kappa_{ia} \kappa_{ja} + \sum_{n=1}^{t} C(n) \left[\sum_{a=1}^{t-n} \kappa_{ia} \kappa_{ja+n} + \sum_{a=n+1}^{t} \kappa_{ia} \kappa_{ja-n} \right] \end{aligned} \tag{8}$$

where we have introduced the function $C(n)$ to denote the average correlation between data points that are n time points apart; i.e.,

$$\begin{aligned} C(0) &= \langle x_a^2 \rangle \\ C(n) &= \langle x_a x_{a+n} \rangle \end{aligned} \tag{9}$$

If we assume that the data come from a change-point process with hazard rate h, then we can compute the form of $C(n)$. If a change-point occurs between time a and time $a+n$, then both x_a and x_{a+n} are sampled from the same generative distribution. In this case $\langle x_a x_{a+n} \rangle$ is simply the mean square of the prior over μ; i.e.,

$$\begin{aligned} \langle x_a x_{a+n} \rangle_{\text{no change-point}} &= \zeta_0 \\ &= \int\int\int x_a x_{a+n} p(x_a|\mu) p(x_{a+n}|\mu) p(\mu|v_p, \chi_p) dx_a dx_{a+n} d\mu \\ &= \int p(\mu|v_p, \chi_p) \mu^2 d\mu \end{aligned} \tag{10}$$

If there is a change-point between time a and time $a+n$ then the parameters of the generating distributions are different. In this case we have that $\langle x_a x_{a+n} \rangle$ is the square mean of the prior; i.e.,

$$\langle x_a x_{a+n} \rangle_{\text{change-point}} = \zeta_1 = \left[\int p(\mu|v_p, \chi_p) \mu d\mu \right]^2 \tag{11}$$

Thus we can write

$$
\begin{aligned}
C(n) &= (1-h)^n \langle x_a x_{a+n} \rangle_{\text{no change-point}} + (1-(1-h)^n) \langle x_a x_{a+n} \rangle_{\text{change-point}} \\
&= (\zeta_0 - \zeta_1)(1-h)^n + \zeta_1
\end{aligned}
\tag{12}
$$

Now, since all of the sums in equation 8 are geometric progressions they can be written in closed form,

$$
\begin{aligned}
\sum_{a=1}^{t-n} \kappa_{ia}\kappa_{ja+n} &= \frac{\alpha_i \alpha_j (1-\alpha_i)^n (1-(1-\alpha_i)^{t-n}(1-\alpha_j)^{t-n})}{1-(1-\alpha_i)(1-\alpha_j)} \\
&= \Theta_{ij}(n)
\end{aligned}
\tag{13}
$$

Note that, by symmetry,

$$
\sum_{a=n+1}^{t} \kappa_{ia}\kappa_{ja-n} = \sum_{a=1}^{t-n} \kappa_{ja}\kappa_{ia+n} = \Theta_{ji}(n) \tag{14}
$$

and we also have

$$
\sum_{a=1}^{t} \kappa_{ia}\kappa_{ja} = \Theta_{ij}(0) \tag{15}
$$

Next the sums over n can be computed as

$$
\begin{aligned}
\sum_{n=1}^{t} C(n)\Theta_{ij}(n) &= \frac{\alpha_i \alpha_j}{1-(1-\alpha_i)(1-\alpha_j)} \left[\sum_{n=1}^{t} C(n)(1-\alpha_i)^n - (1-\alpha_i)^t \sum_{n=1}^{t} C(n)(1-\alpha_j)^{t-n} \right] \\
&= \frac{\alpha_i \alpha_j}{1-(1-\alpha_i)(1-\alpha_j)} (S_i^1 - (1-\alpha_i)^t S_j^2)
\end{aligned}
\tag{16}
$$

where

$$
S_i^1 = \frac{(\zeta_0 - \zeta_1)(1-h)(1-\alpha_i)(1-(1-h)^t(1-\alpha_i)^t)}{1-(1-\alpha_i)(1-h)} + \frac{\zeta_1(1-\alpha_i)(1-(1-\alpha_i)^t)}{\alpha_i} \tag{17}
$$

and

$$
S_j^2 = (\zeta_0 - \zeta_1)(1-h)\frac{(1-\alpha_j)^t - (1-h)^t}{h - \alpha_j} - \frac{\zeta_1((1-\alpha_j)^t - 1)}{\alpha_j} \tag{18}
$$

Which gives us the following expression for $\langle \mu_i \mu_j \rangle$

$$
\begin{aligned}
\langle \mu_i \mu_j \rangle = \frac{\alpha_i \alpha_j (1-(1-\alpha_i)^t(1-\alpha_j)^t)}{1-(1-\alpha_i)(1-\alpha_j)} C(0) &+ \frac{\alpha_i \alpha_j}{1-(1-\alpha_i)(1-\alpha_j)} (S_i^1 - (1-\alpha_i)^t S_j^2) \\
&+ \frac{\alpha_i \alpha_j}{1-(1-\alpha_i)(1-\alpha_j)} (S_j^1 - (1-\alpha_j)^t S_i^2)
\end{aligned}
\tag{19}
$$

Term 4: $\langle p_i \rangle$

$\langle p_i \rangle$ is the average value of the run-length distribution at node i; i.e.,

$$
\langle p_i \rangle = \langle p(l_i | x_{1:t}) \rangle \tag{20}
$$

To compute it, we consider the average of the update equation for the run-length distribution

$$
p(l_i | x_{1:t}) \propto p(x_t | l_i) \sum_{j=1}^{N} p(l_i | l_j) p(l_j | x_{1:t-1}) \tag{21}
$$

Which, for brevity we rewrite as

$$p_i^{t+1} = \frac{\mathcal{L}_i^t \sum_j T_{ij} p_j^t}{\sum_i \mathcal{L}_i^t \sum_j T_{ij} p_j^t} \tag{22}$$

where $p_i^{t+1} = p(l_i|x_{1:t})$, is the probability at time $t+1$, $\mathcal{L}_i^t = p(x_t|l_i)$, is the likelihood of the data for node i at time t and $T_{ij} = p(l_i|l_j)$ is the transition matrix encoding the change-point prior.

We proceed by taking the average of this update over all possible realizations of the data, $x_{1:t}$; i.e.,

$$\begin{aligned} \left\langle p_i^{t+1} \right\rangle &= \left\langle \frac{\mathcal{L}_i^t \sum_j T_{ij} p_j^t}{\sum_i \mathcal{L}_i^t \sum_j T_{ij} p_j^t} \right\rangle \\ &\approx \frac{\langle \mathcal{L}_i^t \rangle \sum_j T_{ij} \langle p_j^t \rangle}{\sum_i \langle \mathcal{L}_i^t \rangle \sum_j T_{ij} \langle p_j^t \rangle} \end{aligned} \tag{23}$$

Where we have made the approximation that the average can be considered separately for $\mathcal{L}_i^t$ and p_i^t.

At equilibrium we have $\langle p_i^t \rangle = \langle p_i^{t+1} \rangle = \langle p_i \rangle$ and $\langle \mathcal{L}_i^t \rangle = \langle \mathcal{L}_i^{t+1} \rangle = \langle \mathcal{L}_i \rangle$ which gives

$$\langle p_i \rangle \propto \langle \mathcal{L}_i \rangle \sum_j T_{ij} \langle p_j \rangle \tag{24}$$

This implies that the mean of run-length distribution is related to the eigenvectors of the matrix, M, where

$$M_{ij} = \langle \mathcal{L}_i \rangle T_{ij} \tag{25}$$

Thus to compute the mean run-length distribution, all that remains is to compute, $\langle \mathcal{L}_i \rangle$. By definition we can write this as

$$\begin{aligned} \langle \mathcal{L}_i \rangle &= \int \int ... \int dx_1 dx_2 ... dx_{t+1} \mathcal{L}_i p(x_{1:t+1}) \\ &= \int dx_{t+1} p(x_{t+1}|\mu_i) \int \int ... \int dx_1 dx_2 ... dx_t p(\mu_i|x_{1:t}, \alpha_i) p(x_{1:t+1}) \end{aligned} \tag{26}$$

where $p(x_{1:t+1})$ is the prior over the data. It will be useful to write this prior distribution in the following form

$$p(x_{1:t+1}) = \sum_r p(r) \int dm \prod_{a=t-r+2}^{t+1} p(x_a|m) \prod_{b=1}^{t-r+1} p(x_b|x_{1:b-1}) \tag{27}$$

This expresses the prior distribution in terms of the time r since the last change-point and the current generative mean m. Substituting this form into 26 gives

$$\langle \mathcal{L}_i \rangle = \sum_r p(r) \int dm \int dx_{t+1} p(x_{t+1}|m) \int d\mu_i p(x_{t+1}|\mu_i) p(\mu_i|m, r) \tag{28}$$

where we have defined $p(\mu_i|m, r)$ as the distribution over the mean of node i given that the mean of the current epoch is m and that the last change-point occurred r time steps ago. This is given by

$$p(\mu_i|m, r) = \int dx_{1:t} p(\mu_i|x_{1:t}, \alpha_i) \prod_{a=t-r+2}^{t+1} p(x_a|m) \prod_{b=1}^{t-r+1} p(x_b|x_{1:b-1}) \tag{29}$$

To get a handle on $p(\mu_i|m, r)$, we compute its moments and use moment matching to get an approximate form. The form will depend on the type of generative distribution. Here we compute it for the Bernoulli and Gaussian cases. In these case we only need the first two moments. Simulations suggest that this approximation is very good in these cases.

First two moments of $p(\mu_i|m,r)$

1st moment For notational convenience later on, we write the mean of $p(\mu_i|m,r)$ as ϕ. This mean is given by

$$\phi = \sum_{a=1}^{t-r} \kappa_a \langle x_a \rangle + \sum_{a=t-r+1}^{t} \kappa_a \langle x_a \rangle \tag{30}$$

where the first term is the sum over times before the last change-point and the second term the sum over times after the last change-point. Before the last change-point $\langle x_a \rangle = m_p$ and after $\langle x_a \rangle = m$. Therefore we have

$$\phi = m_p \left((1-\alpha_i)^r - (1-\alpha_i)^t \right) + m \left(1 - (1-\alpha_i)^r\right) \tag{31}$$

2nd moment As we did above for the derivation of the mean, we break into components before and after the last change-point

$$\begin{aligned} \left\langle \mu_i^2 \right\rangle &= \sum_{a=t-r+1}^{t} \sum_{b=t-r+1}^{t} \kappa_a \kappa_b \langle x_a x_b \rangle + 2 \sum_{a=1}^{t-r} \sum_{b=t-r+1}^{t} \kappa_a \kappa_b \langle x_a x_b \rangle + \sum_{a=1}^{t-r} \sum_{b=1}^{t-r} \kappa_a \kappa_b \langle x_a x_b \rangle \\ &= Q_1 + Q_2 + Q_3 \end{aligned} \tag{32}$$

Now, for $a > t-r$ and $b > t-r$ we have that x_a and x_b are sampled from the same distribution with mean m. Also note that $x_a = x_b$ when $a = b$. Therefore we can write

$$\langle x_a x_b \rangle = \begin{cases} \int\int dx_a dx_b x_a x_b p(x_a|m) p(x_b|m) = m^2 & a \neq b, a > t-r, b > t-r \\ \int dx_a x_a^2 p(x_a|m) = m^2 + \sigma^2 & a = b, a > t-r, b > t-r \end{cases} \tag{33}$$

where σ^2 is the variance of the generative distribution of the data; i.e., the variance of $p(x|m)$. So we have

$$\begin{aligned} Q_1 &= m^2 \sum_{a=t-r+1}^{t} \sum_{b=t-r+1}^{t} \kappa_a \kappa_b + \sigma^2 \sum_{a=t-r+1}^{t} \kappa_a^2 \\ &= m^2 \left(1 - (1-\alpha_i)^r\right)^2 + \frac{\alpha_i^2 \sigma^2 (1 - (1-\alpha_i)^{2r})}{1 - (1-\alpha_i)^2} \end{aligned} \tag{34}$$

For Q_2 we have $a > t-r$ and $b \leq t-r$ which gives

$$\begin{aligned} \langle x_a x_b \rangle &= \int \int dx_a dx_b x_a x_b p(x_a|m) p(x_b|prior) \\ &= m m_p \end{aligned} \tag{35}$$

This gives

$$\begin{aligned} Q_2 &= m m_p \sum_{t-r+1}^{t} \sum_{b=1}^{t-r} \kappa_a \kappa_b \\ &= m m_p \left(1 - (1-\alpha_i)^r\right) \left((1-\alpha_i)^r - (1-\alpha_i)^t \right) \end{aligned} \tag{36}$$

For Q_3 we have

$$Q_3 = \sum_{a=1}^{t-r} \sum_{b=1}^{t-r} \kappa_a \kappa_b \langle x_a x_b \rangle \tag{37}$$

Note that this is equal to Term 3, $\langle \mu_i \mu_j \rangle$, except that, instead of from 1 to t, the sums are instead from 1 to $t-r$. For large t, this allows us to write

$$\begin{aligned} Q_3 &= \sum_{a=1}^{t-r} \sum_{b=1}^{t-r} \kappa_a \kappa_b \langle x_a x_b \rangle \\ &= \sum_{a'=r}^{t} \sum_{b'=r}^{t} \kappa_{a'-r} \kappa_{b'-r} \langle x_{a'-r} x_{b'-r} \rangle \\ &= \sum_{a'=r}^{t} \sum_{b'=r}^{t} (1-\alpha)^{2r} \kappa_{a'} \kappa_{b'} \langle x'_a x'_b \rangle \\ &\approx \sum_{a'=1}^{t} \sum_{b'=1}^{t} (1-\alpha)^{2r} \kappa_{a'} \kappa_{b'} \langle x'_a x'_b \rangle \\ &= (1-\alpha)^{2r} \langle \mu_i \mu_j \rangle \end{aligned} \tag{38}$$

Finally, for notational convenience we define the variance of $p(\mu_i|m,r)$ as ψ^2.

Bernoulli case

For the Bernoulli case, we approximate $p(\mu_i|m,r)$ with a beta distribution of the form

$$p(\mu_i|m,r) \propto \mu_i^{A-1}(1-\mu_i)^{B-1} \tag{39}$$

where parameters A and B that are related to the moments of $p(\mu_i|m,r)$ as

$$\begin{aligned} A &= \frac{\phi(\phi(1-\phi)-\psi^2)}{\psi^2} \\ B &= \frac{(1-\phi)(\phi(1-\phi)-\psi^2)}{\psi^2} \end{aligned} \tag{40}$$

Substituting this into equation 28 for $\langle \mathcal{L}_i \rangle$ gives the following closed form expression

$$\langle \mathcal{L}_i \rangle \approx \frac{(Y-X)h(1-((1-\alpha_i)(1-h))^{t+1})}{1-(1-\alpha_i)(1-h)} + (X+Z-Y(1-\alpha_i)^t)(1-(1-h)^{t+1}) \tag{41}$$

where

$$\begin{aligned} X &= \frac{2(A+1)}{(A+B+1)(A+B)} - \frac{A}{A+B} \\ Y &= \frac{2A}{A+B} - 1 \\ Z &= 1 - \frac{A}{A+B} \end{aligned} \tag{42}$$

Gaussian case

In the Gaussian case, we approximate $p(\mu_i|m,r)$ using a Gaussian distribution with mean ϕ and variance ψ^2. Substituting this into equation 28 we get the following expression for $\mathcal{L}_i$

$$\mathcal{L}_i = \frac{1}{\sqrt{2\pi}} \sum_{r=0}^{T} p(r) \frac{1}{\sqrt{\psi^2 + \sigma_i^2 + \sigma^2 + (1-\alpha_i)^{2r}\sigma_p^2}} \tag{43}$$

where σ is the variance of the generative distribution of the data, $p(x_t|m)$, $\sigma_i = \sigma\sqrt{1+\alpha}$ is the variance of the predictive distribution on node i, $p(x_i|l_i)$, and $\sigma_p = \sigma/\sqrt{v_p}$ is the variance of the prior distribution over the mean $p(m|v_p, \chi_p)$. Finally, $p(r)$ is the prior over the run-length to the last change-point and, for a constant hazard rate, is given by

$$p(r) \propto h(1-h)^r \tag{44}$$

Unlike the Bernoulli case, in which the sum over the run-length, r, can be evaluated exactly, in the Gaussian case we have to evaluate this sum explicitly. For the number of trials used in our task this is straightforward.

Taken together these expressions allow us to compute the average run-length distribution for a given hazard rate and generative distribution.

Figure Legends

Figure S1. Histograms of fit parameter values for all models. Each column represents a model, with the name of the model given at the top. Each row represents a single variable going, in order from top to bottom: hazard rate, decision noise standard deviation, learning rate 1, learning rate 2 and learning rate 3. Where a particular model does not have a particular parameter that box is left empty.

Table Legends

Table S1. Table showing correlation coefficient between simulated and fit parameter values.
