# Supplementary material for: Correction: A Mixture of Delta-Rules Approximation to Bayesian Inference in Change-Point Problems
Source: PLoS Comput Biol. 2018 Jun 26;14(6):e1006210. doi: 10.1371/journal.pcbi.1006210 (PMC6019100; doi:10.1371/journal.pcbi.1006210)
Supplement: S1 Table — (PDF) [file pcbi.1006210.s002.pdf]

Tables

Model	parameter	correlation coefficient	p-value
Full	h	$r = 0.92$	$p = 0$
	σ_d	$r = 1.00$	$p = 0$
Nassar et al.	h	$r = 0.99$	$p = 0$
	σ_d	$r = 1.00$	$p = 0$
1-node	σ_d	$r = 0.99$	$p = 0$
	α_1	$r = 1.00$	$p = 0$
2-node	h	$r = 0.85$	$p =$ 7.48e-18
	σ_d	$r = 0.94$	$p =$ 4.13e-29
	α_1	$r = 0.99$	$p =$ 1.13e-46
	α_2	$r = 0.78$	$p =$ 2.36e-13
3-node	h	$r = 0.67$	$p =$ 4.79e-09
	σ_d	$r = 0.85$	$p =$ 4.34e-18
	α_1	$r = 0.96$	$p =$ 1.57e-34
	α_2	$r = 0.48$	$p =$ 1.24e-04
	α_3	$r = 0.83$	$p =$ 2.84e-16

Figure S 1. Table showing correlation coefficient between simulated and fit parameter values.
