# Supplementary material for: Correction: A Mixture of Delta-Rules Approximation to Bayesian Inference in Change-Point Problems
Source: PLoS Comput Biol. 2018 Jun 26;14(6):e1006210. doi: 10.1371/journal.pcbi.1006210 (PMC6019100; doi:10.1371/journal.pcbi.1006210)
Supplement: S1 Text — (PDF) [file pcbi.1006210.s003.pdf]

Supplementary Material: A mixture of Delta-rules approximation to Bayesian inference in change-point problems

Robert C. Wilson[1,*], Matthew R. Nassar[2], Joshua I. Gold[2]
1 Princeton Neuroscience Institute, Princeton University, Princeton, NJ 08540
2 Department of Neuroscience, University of Pennsylvania, Philadelphia, PA 19104
∗ E-mail: rcw2@princeton.edu

Full derivation of approximate error for one-node case

In order to compute the mean squared error we need expressions for three terms in equation 47 of the main text. These terms are: $\left\langle \left(m^G\right)^2 \right\rangle$, $\langle \mu_1 m^G \rangle$, and $\langle \mu_1^2 \rangle$. We now derive these terms one at a time.

Term 1: $\left\langle \left(m^G\right)^2 \right\rangle$

The simplest of these is just the square mean of the prior distribution over m^G the ground truth mean; i.e.,

$$\left\langle \left(m^G\right)^2 \right\rangle = \int \left(m^G\right)^2 p(m^G|v_p, \chi_p) dm^G \tag{1}$$

This term is defined by our choice of the prior.

Term 2: $\langle \mu_i m^G \rangle$

To compute the second term, $\langle \mu_i m^G \rangle$, we first express the means, μ_i, of the individual Delta rules as weighted sum of all previous data points; i.e.,

$$\begin{aligned} \mu_i &= \sum_{a=1}^{t} \alpha_i (1-\alpha_i)^{t-a} x_a \\ &= \sum_{a=1}^{t} \kappa_{ia} x_a \end{aligned} \tag{2}$$

where the kernel $\kappa_{ia} = \alpha_i(1-\alpha_i)^{t-a}$. Using this kernel expression for μ_i, we can write

$$\langle \mu_i m^G \rangle = \sum_{a=1}^{t} \kappa_{ia} \langle x_a m^G \rangle \tag{3}$$

If there is no change-point between time a and time $t+1$, then x_a is sampled from a distribution with mean m^G and we have

$$\langle x_a m^G \rangle_{\text{no change-point}} = \left\langle \left(m^G\right)^2 \right\rangle \tag{4}$$

which is just the square mean of the prior over m^G. Conversely, if there is a change-point between a and $t+1$, then x_a comes from a different distribution and we have

$$\langle x_a m^G \rangle_{\text{change-point}} = \langle m_p m^G \rangle = m_p \langle m^G \rangle = m_p^2 \tag{5}$$

where m_p is the mean of the prior distribution over m^G.

Finally, to compute $\langle x_a m^G \rangle$ we need to marginalize over the two possibilities that a change-point has occurred or not. The probability that there is no change-point between times a and $t+1$ is $(1-h)^{t-a+1}$ and a change happens with probability $1-(1-h)^{t-a+1}$. These probabilities give us the following expression for $\langle x_a m^G \rangle$,

$$\begin{aligned}\langle x_a m^G \rangle &= (1-h)^{t-a+1} \langle x_a m^G \rangle_{\text{no change-point}} + \left(1-(1-h)^{t-a+1}\right) \langle x_a m^G \rangle_{\text{change-point}} \\ &= (1-h)^{n+1} \left(\left\langle \left(m^G\right)^2 \right\rangle - m_p^2 \right) + m_p^2 \\ &= (1-h)^{n+1} \xi_0 + \xi_1\end{aligned} \tag{6}$$

where we have defined $n = t-a$, $\xi_0 = \left\langle \left(m^G\right)^2 \right\rangle - m_p^2$ and $\xi_1 = m_p^2$. Thus we can write

$$\begin{aligned}\langle \mu_i m^G \rangle &= \sum_{n=0}^{t-1} \alpha_i (1-\alpha_i)^n \left(\xi_0 (1-h)^{n+1} + \xi_1 \right) \\ &= \frac{\xi_0 \alpha_i (1-h)\left(1-(1-\alpha_i)^t (1-h)^t\right)}{1-(1-\alpha_i)(1-h)} + \xi_1 \left(1-(1-\alpha_i)^t\right)\end{aligned} \tag{7}$$

Term 3: $\langle \mu_i \mu_j \rangle$

For completeness, we consider the general case of average of two means, μ_i and μ_j, generated with two separate learning rates. The specific case, $\langle \mu_1^2 \rangle$ is easily computed from this by setting $i = j = 1$.

$\langle \mu_i \mu_j \rangle$, is calculated in a similar manner to $\langle \mu_i m^G \rangle$. Using the kernel expression for μ_i (equation 2), we can write

$$\begin{aligned}\langle \mu_i \mu_j \rangle &= \sum_{a=1}^{t} \sum_{b=1}^{t} \kappa_{ia} \kappa_{jb} \langle x_a x_b \rangle \\ &= C(0) \sum_{a=1}^{t} \kappa_{ia} \kappa_{ja} + \sum_{n=1}^{t} C(n) \left[\sum_{a=1}^{t-n} \kappa_{ia} \kappa_{ja+n} + \sum_{a=n+1}^{t} \kappa_{ia} \kappa_{ja-n} \right]\end{aligned} \tag{8}$$

where we have introduced the function $C(n)$ to denote the average correlation between data points that are n time points apart; i.e.,

$$\begin{aligned}C(0) &= \langle x_a^2 \rangle \\ C(n) &= \langle x_a x_{a+n} \rangle\end{aligned} \tag{9}$$

If we assume that the data come from a change-point process with hazard rate h, then we can compute the form of $C(n)$. If a change-point occurs between time a and time $a+n$, then both x_a and x_{a+n} are sampled from the same generative distribution. In this case $\langle x_a x_{a+n} \rangle$ is simply the mean square of the prior over μ; i.e.,

$$\begin{aligned}\langle x_a x_{a+n} \rangle_{\text{no change-point}} &= \zeta_0 \\ &= \int \int \int x_a x_{a+n} p(x_a|\mu) p(x_{a+n}|\mu) p(\mu|v_p, \chi_p) dx_a dx_{a+n} d\mu \\ &= \int p(\mu|v_p, \chi_p) \mu^2 d\mu\end{aligned} \tag{10}$$

If there is a change-point between time a and time $a+n$ then the parameters of the generating distributions are different. In this case we have that $\langle x_a x_{a+n} \rangle$ is the square mean of the prior; i.e.,

$$\langle x_a x_{a+n} \rangle_{\text{change-point}} = \zeta_1 = \left[\int p(\mu|v_p, \chi_p) \mu d\mu \right]^2 \tag{11}$$

Thus we can write

$$\begin{aligned} C(n) &= (1-h)^n \langle x_a x_{a+n} \rangle_{\text{no change-point}} + (1-(1-h)^n) \langle x_a x_{a+n} \rangle_{\text{change-point}} \\ &= (\zeta_0 - \zeta_1)(1-h)^n + \zeta_1 \end{aligned} \tag{12}$$

Now, since all of the sums in equation 8 are geometric progressions they can be written in closed form,

$$\begin{aligned} \sum_{a=1}^{t-n} \kappa_{ia}\kappa_{ja+n} &= \frac{\alpha_i\alpha_j(1-\alpha_i)^n(1-(1-\alpha_i)^{t-n}(1-\alpha_j)^{t-n})}{1-(1-\alpha_i)(1-\alpha_j)} \\ &= \Theta_{ij}(n) \end{aligned} \tag{13}$$

Note that, by symmetry,

$$\sum_{a=n+1}^{t} \kappa_{ia}\kappa_{ja-n} = \sum_{a=1}^{t-n} \kappa_{ja}\kappa_{ia+n} = \Theta_{ji}(n) \tag{14}$$

and we also have

$$\sum_{a=1}^{t} \kappa_{ia}\kappa_{ja} = \Theta_{ij}(0) \tag{15}$$

Next the sums over n can be computed as

$$\begin{aligned} \sum_{n=1}^{t} C(n)\Theta_{ij}(n) &= \frac{\alpha_i\alpha_j}{1-(1-\alpha_i)(1-\alpha_j)} \left[\sum_{n=1}^{t} C(n)(1-\alpha_i)^n - (1-\alpha_i)^t \sum_{n=1}^{t} C(n)(1-\alpha_j)^{t-n} \right] \\ &= \frac{\alpha_i\alpha_j}{1-(1-\alpha_i)(1-\alpha_j)} (S_i^1 - (1-\alpha_i)^t S_j^2) \end{aligned} \tag{16}$$

where

$$S_i^1 = \frac{(\zeta_0-\zeta_1)(1-h)(1-\alpha_i)(1-(1-h)^t(1-\alpha_i)^t)}{1-(1-\alpha_i)(1-h)} + \frac{\zeta_1(1-\alpha_i)(1-(1-\alpha_i)^t)}{\alpha_i} \tag{17}$$

and

$$S_j^2 = (\zeta_0-\zeta_1)(1-h)\frac{(1-\alpha_j)^t-(1-h)^t}{h-\alpha_j} - \frac{\zeta_1((1-\alpha_j)^t-1)}{\alpha_j} \tag{18}$$

Which gives us the following expression for $\langle \mu_i \mu_j \rangle$

$$\begin{aligned} \langle \mu_i\mu_j \rangle = \frac{\alpha_i\alpha_j(1-(1-\alpha_i)^t(1-\alpha_j)^t)}{1-(1-\alpha_i)(1-\alpha_j)} C(0) &+ \frac{\alpha_i\alpha_j}{1-(1-\alpha_i)(1-\alpha_j)} (S_i^1 - (1-\alpha_i)^t S_j^2) \\ &+ \frac{\alpha_i\alpha_j}{1-(1-\alpha_i)(1-\alpha_j)} (S_j^1 - (1-\alpha_j)^t S_i^2) \end{aligned} \tag{19}$$
