# Supplementary material for: Correction: A Mixture of Delta-Rules Approximation to Bayesian Inference in Change-Point Problems
Source: PLoS Comput Biol. 2018 Jun 26;14(6):e1006210. doi: 10.1371/journal.pcbi.1006210 (PMC6019100; doi:10.1371/journal.pcbi.1006210)
Supplement: S2 Text — (PDF) [file pcbi.1006210.s004.pdf]

A mixture of Delta-rules approximation to Bayesian inference in change-point problems

Robert C. Wilson[1,*], Matthew R. Nassar[2], Gaia Tavoni[2], Joshua I. Gold[2]
1 Department of Psychology and Cognitive Science Program, University of Arizona, Tucson AZ 85721
2 Department of Neuroscience, University of Pennsylvania, Philadelphia, PA 19104
∗ E-mail: bob@arizona.edu

Abstract

Error-driven learning rules have received considerable attention because of their close relationships to both optimal theory and neurobiological mechanisms. However, basic forms of these rules are effective under only a restricted set of conditions in which the environment is stable. Recent studies have defined optimal solutions to learning problems in more general, potentially unstable, environments, but the relevance of these complex mathematical solutions to how the brain solves these problems remains unclear. Here we show that one such Bayesian solution can be approximated by a computationally straightforward mixture of simple error-driven 'Delta' rules. This simpler model can make effective inferences in a dynamic environment and matches human performance on a predictive-inference task using a mixture of a small number of Delta rules. This model represents an important conceptual advance in our understanding of how the brain can use relatively simple computations to make nearly optimal inferences in a dynamic world.

Author Summary

The ability to make accurate predictions is important to thrive in a dynamic world. Many predictions, like those made by a stock picker, are based, at least in part, on historical data thought also to reflect future trends. However, when unexpected changes occur, like an abrupt change in the value of a company that affects its stock price, the past can become irrelevant and we must rapidly update our beliefs. Previous research has shown that, under certain conditions, human predictions are similar to those of mathematical, ideal-observer models that make accurate predictions in the presence of change-points. Despite this progress, these models require superhuman feats of memory and computation and thus are unlikely to be implemented directly in the brain. In this work, we address this conundrum by developing an approximation to the ideal-observer model that drastically reduces the computational load with only a minimal cost in performance. We show that this model better explains human behavior than other models, including the optimal model, and suggest it as a biologically plausible model for learning and prediction.

Introduction

Decisions are often guided by beliefs about the probability and utility of potential outcomes. These beliefs are learned through past experiences that, in stable environments, can be used to generate accurate predictions. However, in dynamic environments, changes can occur that render past experiences irrelevant for predicting future outcomes. For example, after a change in government, historical tax rates may no longer be a reliable predictor of future tax rates. Thus, an important challenge faced by a decision-maker is to identify and respond to environmental change-points, corresponding to when previous beliefs should be abandoned and new beliefs should be formed.

A toy example of such a situation is shown in figure 1A, where we plot the price of a fictional stock over time. In this example, the stock price on a given day (red dots) is generated by sampling from a Gaussian distribution with a standard deviation of $2 and a mean (dashed black line) that starts at $10 before changing abruptly to $20 at a change-point, perhaps caused by the favorable resolution of a court case. A trader only sees the stock price and not the underlying mean but has to make predictions about the stock price on the next day.

One common strategy for computing this prediction is based on the Delta rule:

$$
\begin{aligned}
\delta_t &= x_t - \mu_t \\
\mu_{t+1} &= \mu_t + \alpha \delta_t
\end{aligned}
\tag{1}
$$

According to this rule, an observation, x_t, is used to update an existing prediction, μ_t, based on the learning rate, α and the prediction error, δ_t. Despite its simplicity, this learning rule can provide effective solutions to a wide range of machine-learning problems [1, 2]. In certain forms, it can also account for numerous behavioral findings that are thought to depend on prediction-error signals represented in brainstem dopaminergic neurons, their inputs from the lateral habenula, and their targets in the basal ganglia and the anterior cingulate cortex [3–15].

Unfortunately, this rule does not perform particularly well in the presence of change-points. We illustrate this problem with a toy example in figure 1B and C. In panel B, we plot the predictions of this model for the toy data set when α is set to 0.2. In this case, the algorithm does an excellent job of computing the mean stock value before the change-point. However, it takes a long time to adjust its predictions after the change-point, undervaluing the stock for several days. In figure 1C, we plot the predictions of the model when $\alpha = 0.8$. In this case, the model responds rapidly to the change-point but has larger errors during periods of stability.

One way around this problem is to dynamically update the learning rate on a trial-by-trial basis between zero, indicating that no weight is given to the last observed outcome, and one, indicating that the prediction is equal to the last outcome [16, 17]. During periods of stability, a decreasing learning rate can match the current belief to the average

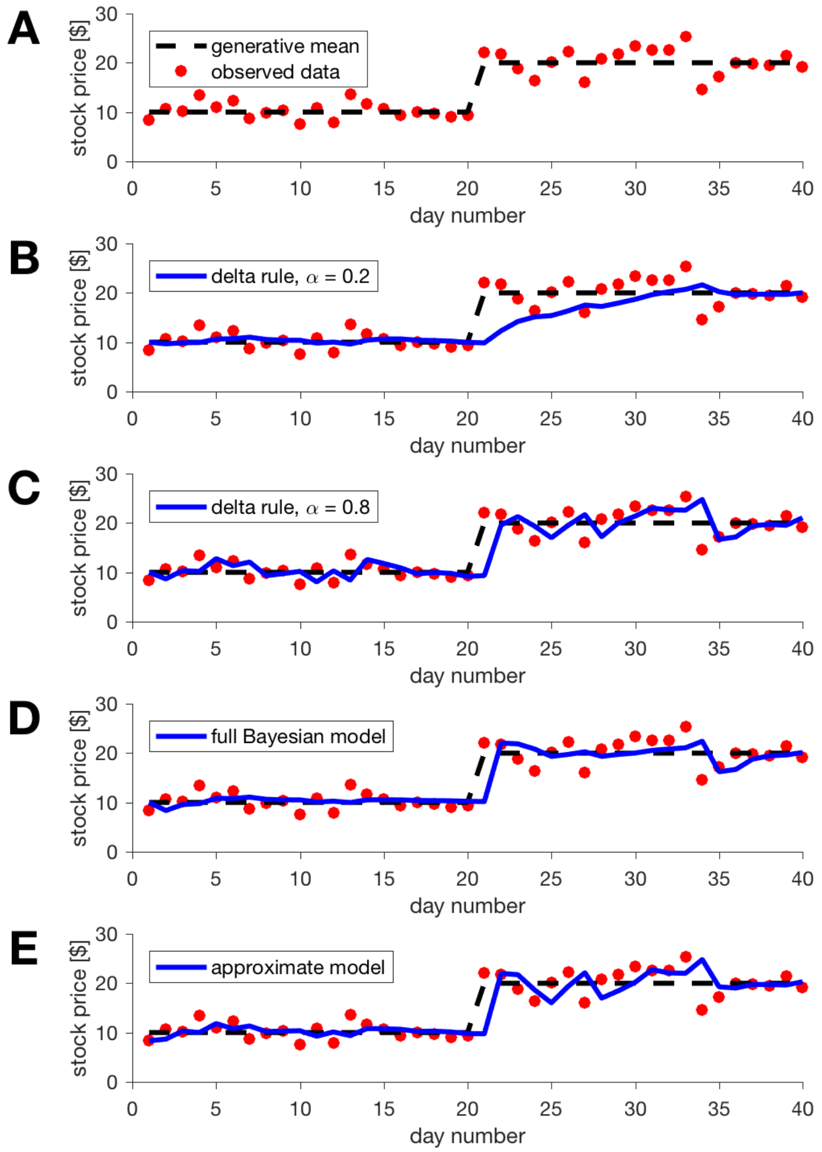

Figure 1. An example change-point problem with a single change-point at time 20 (A) and an illustration of the performance of different algorithms at making predictions (B-E). (B) The Delta rule model with learning rate parameter $\alpha = 0.2$ performs well before the change-point but poorly immediately afterwards. (C) The Delta rule model with learning rate $\alpha = 0.8$ responds quickly to the change-point but has noisier estimates overall. (D) The full Bayesian model dynamically adapts its learning rate to minimize error overall. (E) Our approximate model shows similar performance to the Bayesian model but is implemented at a fraction of the computational cost and in a biologically plausible manner.

outcome. After change-points, a high learning rate shifts beliefs away from historical data and towards more recent, and more relevant, outcomes.

These adaptive dynamics are captured by Bayesian ideal-observer models that determine the rate of learning based on the statistics of change-points and the observed data [18–20]. An example of the behavior of the Bayesian model is shown in figure 1D. In this case, the model uses a low learning rate in periods of stability to make predictions that are very close to the mean, then changes to a high learning rate after a change-point to adapt more quickly to the new circumstances.

Recent experimental work has shown that human subjects adaptively adjust learning rates in dynamic environments in a manner that is qualitatively consistent with these algorithms [16, 17, 21]. However, it is unlikely that subjects are basing these adjustments on a direct neural implementation of the Bayesian algorithms, which are complex and computationally demanding. Thus, in this paper we ask two questions: 1) Is there a simpler, general algorithm capable of adaptively adjusting its learning rate in the presence of change-points? And 2) Does the new model better explain human behavioral data than either the full Bayesian model or a simple Delta rule? We address these questions by developing a simple approximation to the full Bayesian model (figure 1E). In contrast to earlier work that used a single Delta rule with an adaptive learning rate [17,21], our model uses a mixture of biologically plausible Delta rules, each with its own, fixed learning rate, to adapt its behavior in the presence of change-points. We show that the model provides a better match to human performance than the other models. We conclude with a discussion of the biological plausibility of our model, which we propose as a general model of human learning.

Methods

Ethics statement

Human subject protocols were approved by the University of Pennsylvania internal review board. Informed consent was given by all participants prior to taking part in the study.

Change-point processes

To familiarize readers with change-point processes and the Bayesian model, we first review these topics in some detail and then turn our attention to the reduced model.

In this paper we are concerned with data generated from change-point processes. An example of such a process generating Gaussian data is given in figure 2. We start by defining a hazard rate, h, that in the general case can be variable over time but for our purposes is assumed to be constant. Change-point locations are then generated by sampling from a Bernoulli distribution with this hazard rate, such that the probability of a change-point occurring at time t is h (figure 2A). In between change-points, in periods we

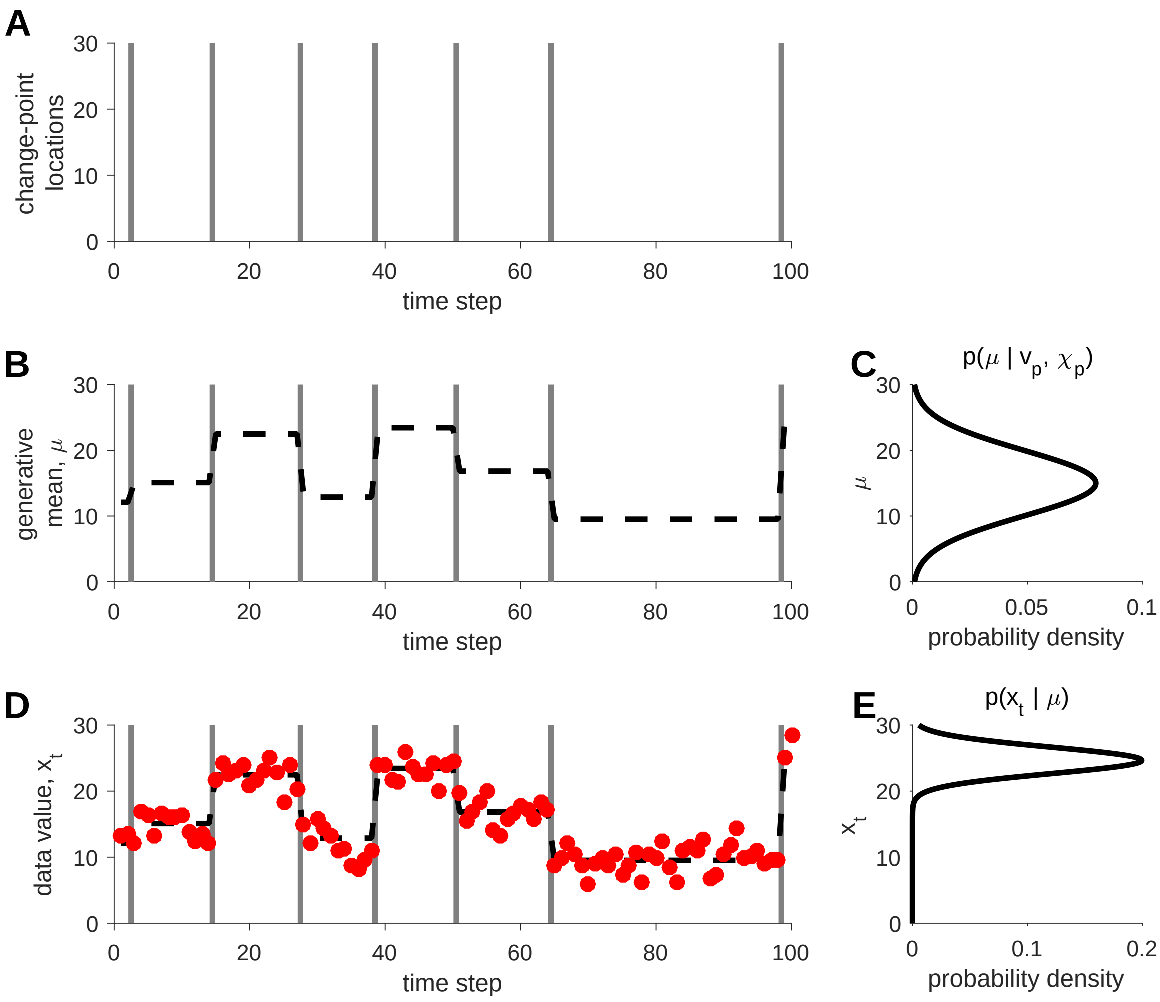

Figure 2. An example of the generative process behind a change-point data set with Gaussian data. (A) First, the change-point locations (grey lines) are sampled from a Bernoulli process with known hazard rate h (in this case, $h = 0.05$). (B) Next, the mean of the Gaussian distribution, μ, is sampled from the prior distribution defined by parameters v_p and χ_p, $p(\mu|v_p, \chi_p)$, (C) for each epoch between change-points (in this case, $v_p = 0.16$ and $\chi_p = 2.4$). (D) Finally, the data points at each time step (x_t) are sampled from a Gaussian distribution with the current mean and a variance of 1, $p(x_t|\mu)$, shown in (E) for the mean of the last epoch.

term 'epochs,' the generative parameters of the data are constant. Within each epoch, the values of the generative parameters, η, are sampled from a prior distribution $p(\eta|v_p, \chi_p)$, for some hyper-parameters v_p and χ_p that will be described in more detail in the following sections. For the Gaussian example, η is simply the mean of the Gaussian at each time point, μ. We generate this mean for each epoch (figure 2B) by sampling from the prior distribution shown in figure 2C. Finally, we sample the data points at each time t, x_t from the generative distribution $p(x_t|\eta)$ (figure 2D and E).

Full Bayesian model

The goal of the full Bayesian model [18,19] is to make accurate predictions in the presence of change-points. This model infers the predictive distribution, $p(x_{t+1}|x_{1:t})$, over the next data point, x_{t+1}, given the data observed up to time t, $x_{1:t} = \{x_1, x_2, ..., x_t\}$.

In the case where the change-point locations are known, computing the predictive distribution is straightforward. In particular, because the parameters of the generative distribution are resampled independently at a change-point (more technically, the change-points separate the data into product partitions [22]) only data seen since the last change-point are relevant for predicting the future. Therefore, if we define the run-length at time t, r_t, as the number of time steps since the last change-point, we can write

$$p(x_{t+1}|x_{1:t}) = p(x_{t+1}|x_{t+1-r_{t+1}:t}) = p(x_{t+1}|r_{t+1}) \tag{2}$$

where we have introduced the shorthand $p(x_{t+1}|r_{t+1})$ to denote the predictive distribution given the last r_{t+1} time points. Assuming that our generative distribution is parameterized by parameters η, then $p(x_{t+1}|r_{t+1})$ is straightforward to write down (at least formally) as the marginal over η

$$p(x_{t+1}|r_{t+1}) = \int p(x_{t+1}|\eta)p(\eta|r_{t+1})d\eta \tag{3}$$

where $p(\eta|r_t) = p(\eta|x_{t-r_t+1:t})$ is the inferred distribution over η given the last r_t time points, and $p(x_t|\eta)$ is the likelihood of the data given the generative parameters.

When the change-point locations are unknown the situation is more complex. In this case we need to compute a probability distribution over all possible values for the run-length given the observed data. This distribution is called the run-length distribution $p(r_t|x_{1:t})$. Once we have the run-length distribution, we can compute the predictive distribution in the following way. First we compute the expected run-length on the next trial, $t + 1$; i.e.,

$$p(r_{t+1}|x_{1:t}) = \sum_{r_t=1}^{t} p(r_{t+1}|r_t)p(r_t|x_{1:t}) \tag{4}$$

where the sum is over all possible values of the run-length at time t and $p(r_{t+1}|r_t)$ is the change-point prior that describes the dynamics of the run-length over time. In particular,

because the run-length either increases by one, with probability $1-h$ in between change-points, or decreases to zero, with probability h at a change-point, the change-point prior, $p(r_{t+1}|r_t)$, takes the following form

$$p(r_{t+1}|r_t) = \begin{cases} 1-h & \text{if } r_{t+1} = r_t + 1 \\ h & \text{if } r_{t+1} = 0 \\ 0 & \text{otherwise} \end{cases} \tag{5}$$

Given the distribution $p(r_{t+1}|x_{1:t})$, we can then compute the predictive distribution of the data on the next trial, $p(x_{t+1}|x_{1:t})$ in the following manner,

$$p(x_{t+1}|x_{1:t}) = \sum_{r_{t+1}=0}^{t+1} p(x_{t+1}|r_{t+1})p(r_{t+1}|x_{1:t}) \tag{6}$$

where the sum is over all possible values of the run-length at time $t+1$.

All that then remains is to compute the run-length distribution itself, which can be done recursively using Bayes' rule

$$\begin{aligned} p(r_t|x_{1:t}) &\propto p(x_t|r_t)p(r_t|x_{1:t-1}) \\ &= p(x_t|r_t) \sum_{r_{t-1}=0}^{t-1} p(r_t|r_{t-1})p(r_{t-1}|x_{1:t-1}) \end{aligned} \tag{7}$$

Substituting in the form of the change-point prior for $p(r_t|r_{t-1})$ we get

$$p(r_t|x_{1:t}) \propto \begin{cases} (1-h)p(x_t|r_t)p(r_{t-1} = r_t - 1|x_{1:t-1}) & \text{if } r_t > 0 \\ hp(x_t|0) & \text{if } r_t = 0 \end{cases} \tag{8}$$

Thus for each value of the run-length, all but two of the of the terms in equation 7 vanish and the algorithm has complexity of $O(t)$ computations per timestep. Unfortunately, although this is a substantial improvement compared to $O(2^t)$ complexity of a more naïve change-point model, this computation is still quite demanding. In principle, the total number of run-lengths we must consider is infinite, because we must allow for the possibility that a change-point occurred at any time in the past. In practice, however, it is usual to introduce a maximum run-length, r_{max}, and define the change-point prior here to be

$$p(r_{t+1}|r_t = r_{max}) = \begin{cases} 1-h & \text{if } r_{t+1} = r_{max} \\ h & \text{if } r_{t+1} = 0 \\ 0 & \text{otherwise} \end{cases} \tag{9}$$

With this procedure, the complexity of the computation is bounded but still can remain dauntingly high.

Efficient solution for exponential families

The above inference algorithm is particularly well suited to problems that involve exponential family distributions (such as the Gaussian, Bernoulli, or Laplace distributions) with a conjugate prior [23]. For these cases, the predictive distribution given the run-length, $p(x_t|r_t)$, can be represented with a finite number of parameters, called sufficient statistics, that are easily updated when new data arrive.

Specifically, we assume that x_t is sampled from a distribution with parameters η, $p(x_t|\eta)$, which can be related to $p(x_t|r_t)$ as

$$p(x_t|r_t) = \int d\eta p(x_t|\eta)p(\eta|r_t) \tag{10}$$

If $p(x_t|\eta)$ is an exponential family distribution and we assume a conjugate prior, then this equation is relatively straightforward to compute. Specifically we assume that $p(x_t|\eta)$ has the form

$$p(x|\eta) = H(x)\exp\left(\eta^T U(x) - A(\eta)\right) \tag{11}$$

where the forms of η, $H(x)$, $U(x)$ and $A(\eta)$ determine the specific type of exponential family distribution. For example, for a Gaussian distribution with unknown mean, μ, and known variance σ, we have

$$\eta = \frac{\mu}{\sigma^2}; \quad H(x) = \frac{1}{\sqrt{2\pi}\sigma}\exp\left(-\frac{x^2}{2\sigma^2}\right); \quad U(x) = x; \quad A(\eta) = \frac{\mu^2}{2\sigma^2} \tag{12}$$

We further assume that the generative parameters, η, are resampled at each change-point from a conjugate prior distribution of the form

$$p(\eta|v_p, \chi_p) = \tilde{H}(\eta)\exp\left(\eta^T \chi_p - v_p A(\eta) - \tilde{A}(v_p, \chi_p)\right) \tag{13}$$

where χ_p and v_p are the prior hyperparameters and the forms of $\tilde{H}(\eta)$ and $\tilde{A}(v_p, \chi_p)$ determine the nature of the prior distribution.

For example, for a Gaussian prior distribution over μ with standard deviation $\sigma/\sqrt{v_p}$ and mean χ_p/v_p, we set

$$\tilde{H}(\eta) = \frac{1}{\sqrt{2\pi}}; \quad \tilde{A}(v_p, \chi_p) = \log\left(\frac{\sigma}{\sqrt{v_p}}\right) + \frac{\chi_p^2}{2v_p\sigma^2}; \tag{14}$$

With this conjugate prior, the posterior distribution over the parameters given the last r_t data points, $p(\eta|r_t)$, has the same form as the prior, $p(\eta|v_p, \chi_p)$ and we can write

$$\begin{aligned} p(\eta|r_t) &= p(\eta|v_t^{r_t}, \chi_t^{r_t}) \\ &= \tilde{H}(\eta)\exp\left(\eta^T \chi_t^{r_t} - v_t^{r_t} A(\eta) - \tilde{A}(v_t^{r_t}, \chi_t^{r_t})\right) \end{aligned} \tag{15}$$

This posterior distribution, $p(\eta|r_t)$ (and thus also the likelihood $p(x_t|r_t)$ by equation 10), is parameterized by the sufficient statistics $v_t^{r_t}$ and $\chi_t^{r_t}$. Crucially, these statistics are straightforward to compute, as follows

$$v_t^{r_t} = r_t + v_p \tag{16}$$

and

$$\chi_t^{r_t} = \chi_p + \sum_{i=t-r_t+1}^{t} U(x_i) \tag{17}$$

Thus, $v_t^{r_t}$ is constant for a given run-length, and $\chi_t^{r_t}$ computes a running sum of the most recent r_t data points (transformed by function U).

It is useful to write the equation for $\chi_t^{r_t}$ as an update rule; that is, in terms of the sufficient statistics at an earlier time point. In particular, for $r_t > 1$, we can write the update in terms of the sufficient statistic at the previous time point and run-length; i.e.,

$$\chi_t^{r_t} = \chi_{t-1}^{r_t - 1} + U(x_t) \tag{18}$$

Dividing through by $v_t^{r_t}$ gives a Delta-rule update for the mean, $\mu_t^{r_t} = \chi_t^{r_t}/v_t^{r_t}$:

$$\mu_t^{r_t} = \mu_{t-1}^{r_t - 1} + \frac{1}{v_{t-1}^{r_t - 1} + 1}\left(U(x_t) - \mu_{t-1}^{r_t - 1}\right) \tag{19}$$

Note that in this case the learning rate, $1/(v_{t-1}^{r_t - 1} + 1)$, decays as the run-length increases.

Graphical interpretation

The previous sections showed that, for conjugate exponential distributions, the Bayesian model needs to keep track of only the run-length distribution, $p(r_t|x_{1:t})$, and the sufficient statistics, $v_t^{r_t}$ and $\chi_t^{r_t}$, for each run-length to fully compute the predictive distribution, $p(x_{t+1}|x_{1:t})$. This algorithm also has an intuitive interpretation in terms of message passing on a graph (Figure 3A). Each node in this graph represents a run-length, r_i, with two properties: 1) the sufficient statistics, $v_t^{r_t}$ and $\chi_t^{r_t}$, associated with that run-length, and 2) a 'weight' representing the probability that the run-length at time t is r_i; i.e., $p(r_t = r_i|x_{1:t})$. The weights of the nodes are computed by passing messages along the edges of the graph. Specifically, each node, r_i, sends out two messages: an 'increasing' message to node $r_i + 1$ that corresponds to an increase in run-length if no change-point occurred, $(1-h)p(r_t|x_{1:t})$, and 2) a 'change-point' message, to r_1, corresponding to a decrease in run-length at a change-point, $hp(r_t|x_{1:t})$. The weight of node r_i is then updated by summing all of the incoming messages and multiplying it by $p(x_{t+1}|r_i)$, which implements equation 8.

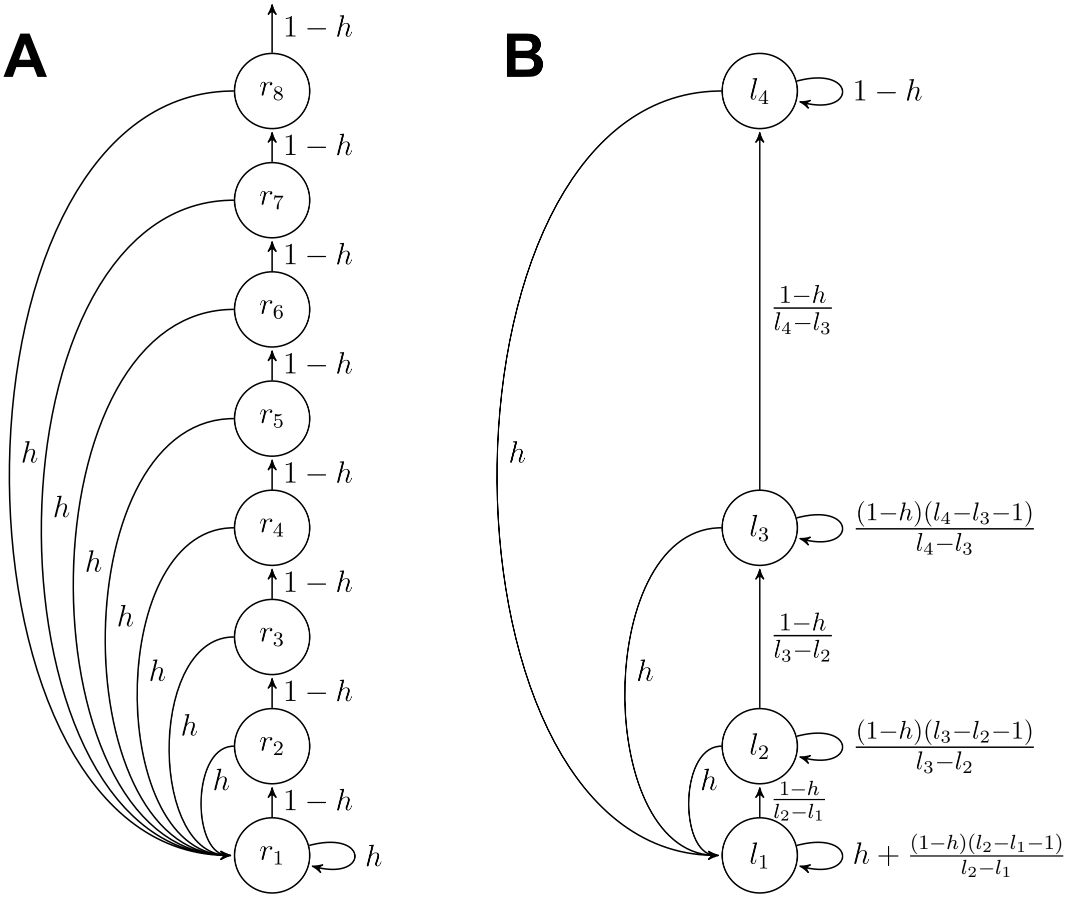

Figure 3. Schematic of the message passing algorithm for the full (A) and approximate (B) algorithms. For the approximate algorithm we only show the case for $l_{i+1} \geq l_i + 1$.

Reduced model

Despite the elegance of the full Bayesian algorithm, it is complex, requiring a memory of a large number (r_{max}) of different run-lengths, which, in the worst case, is equivalent to keeping track of all the past data. Thus, it seems an unlikely model of human cognition, and a key question is whether comparable predictive performance can be achieved with a simpler, more biologically plausible algorithm. Here we introduce an approximation to the full model that addresses these issues. First we reduce the model's complexity by removing nodes from the update graph (Figure 3). Then we transform the update equation for $\chi_t^{r_t}$ into a Delta-rule update equation in which the sufficient statistic on each node updates independently of the other nodes. The resulting algorithm is a biologically plausible mixture of Delta-rules that is able to flexibly adapt its overall learning rate in the presence of change-points and whose performance is comparable with that of the full Bayesian model at a fraction of the computational cost. Below we derive new update equations for the sufficient statistics and the weights of each new node for this reduced model.

To more easily distinguish the full and reduced models, we use l to denote run-length in the reduced model and r to denote run-length in the full model. Thus, the reduced model has N nodes, where node i has run-length l_i. The set of run-lengths, $\{l_i\}$, are ordered such that $l_{i-1} < l_i < l_{i+1}$. Unlike the full model, the run-lengths in the reduced model can take on non-integer values, which allows greater flexibility.

The first step in our approximation is to remove nodes from the update graph. This step reduces the memory demands of the algorithm but also requires us to change the update rule for the sufficient statistic and the form of the change-point prior.

Consider a node with run-length l_i. In the full Bayesian model, the sufficient statistic for this node would be

$$\chi_t^{l_i} = \chi_p + \sum_{t'=t-l_i+1}^{t} U(x_{t'}) = \chi_{t-1}^{l_i-1} + U(x_t) \tag{20}$$

Note that this form of the update relies on having computed $\chi_{t-1}^{l_i-1}$, which is the sufficient statistic at run length $l_i - 1$. In the full Bayesian model, this procedure is straightforward because all possible run-lengths are represented. In contrast, the reduced model includes only a subset of possible run-lengths, and thus a node with run-length $l_i - 1$ will not exist for some values of l_i. Therefore, the reduced model must include a new method for updating the sufficient statistic and a new form of the change-point prior.

We first note that another way of writing the update for $\chi_t^{l_i}$ is as

$$\chi_t^{l_i} = \chi_{t-1}^{l_i} + U(x_t) - U(x_{t-l_i}) \tag{21}$$

This sliding-window update equation depends only on information available at node l_i and thus does not rely on knowing the sufficient statistic at node $l_i - 1$. However, this

update also has a high memory demand because, to update the sliding window, we have to subtract $U(x_{t-l_i})$, which we can only do if we keep track of the previous l_i data points on each node.

In our model, we remove the dependence on x_{t-l_i}, and hence the additional memory demands, by taking the average of equation 21. This procedure leads to a memoryless (yet approximate) form of the update equation for each node. In particular, if we take the average of equation 21 with respect to x_{t-l_i}, we have

$$\begin{aligned} \left\langle \chi_t^{l_i} \right\rangle_{x_{t-l_i}} &= \left\langle \chi_{t-1}^{l_i} \right\rangle_{x_{t-l_i}} + U(x_t) - \left\langle U(x_{t-l_i}) \right\rangle_{x_{t-l_i}} \\ &\approx \hat{\chi}_{t-1}^{l_i} + U(x_t) - \mu_{t-1}^{l_i} \end{aligned} \tag{22}$$

where we have introduced $\hat{\chi}_t^{l_i} \approx \left\langle \chi_t^{l_i} \right\rangle_{x_{t-l_i}}$ as the Delta-rule's approximation to the mean sufficient statistic and

$$\mu_t^{l_i} = \frac{\hat{\chi}_t^{l_i}}{l_i + v_p} \tag{23}$$

as the mean of the node. Dividing equation 21 by $l_i + v_p$ gives us the following form of the update for the mean

$$\mu_t^{l_i} = \mu_{t-1}^{l_i} + \frac{1}{l_i + v_p} \left(U(x_t) - \mu_{t-1}^{l_i} \right) \tag{24}$$

Note that this equation for the update of $\mu_t^{l_i}$ is a Delta rule, just like equation 1, with a fixed learning rate, $\alpha_i = 1/(l_i + v_p)$. Thus, the reduced model simply has to keep track of $\mu_t^{l_i}$ for each node and update it using only the most recent data point. This form of update rule also allows us to interpret non-integer values of the run-length, l_i, in terms of changes in the learning rate of the Delta rule on a continuum. In figure 4 we show the effect of this approximation on the extent to which past data points are used to compute the mean of each node. The sliding window rule computes the average across the last l_i data points, ignoring all previous data. In contrast, the Delta rule computes a weighted average using an exponential that decays over time, which tends to slightly under-emphasize the contributions of recent data and over-emphasize the contributions of distant data relative to the sliding window.

Reducing the number of nodes in the model also requires us to change how we update the weights of each node. In particular the update for the weights, $p(l_i|x_{1:t})$, is given as

$$p(l_i|x_{1:t}) \propto p(x_t|l_i) \sum_{j=1}^{N} p(l_i|l_j) p(l_j|x_{1:t-1}) \tag{25}$$

This equation is similar to equation 7 but differs in the number of run-lengths available. Crucially, this difference requires an adjustment to the change-point prior. The adjusted prior should approximate the full change-point prior (Eq. 5) as closely as possible. Recall

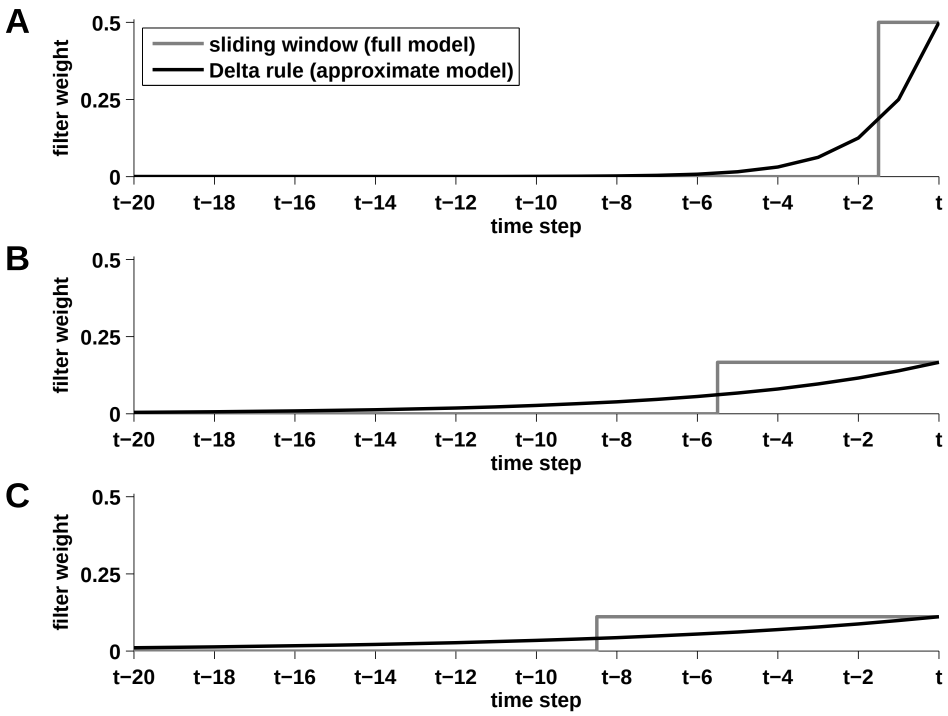

Figure 4. Comparison of the extent to which the sliding window and Delta rule updates weigh past information in computing the mean of a node for three different run-lengths: (A) $l_i = 2$, (B) $l_i = 6$ and (C) $l_i = 9$.

that the full prior captures the fact that the run-length either decreases to zero if there is a change-point (with prior probability h) or increases by one if there is no change-point (with prior probability $1 - h$).

To see how to compute this adjusted prior in the reduced model, we first decompose the change-point prior into two terms corresponding to the possibility that a change-point will occur or not; i.e.,

$$p(l_i|l_j) = hp(l_i|l_j, \text{change}) + (1 - h)p(l_i|l_j, \text{no change}) \tag{26}$$

where $p(l_i|l_j, \text{change})$ is the probability that the run-length is l_i given that there was a change-point and that the previous run-length was l_j. Similarly $p(l_i|l_j, \text{no change})$ is the probability that the run-length is l_i given that the previous run-length was l_j and there was not a change-point.

The change-point case is straightforward, because a change-point always results in a transition to the shortest run-length; i.e., $p(l_i|l_j, \text{change})$ is zero, except when $i = 1$ when it takes value 1.

The no change-point case, however, is more difficult. In the full model the run-length

increases by 1 when there is no change-point, thus we would like to have

$$p(l_i|l_j, \text{no change}) = \begin{cases} 1 & \text{if } l_i = l_j + 1 \\ 0 & \text{otherwise} \end{cases} \tag{27}$$

However, because the nodes have variable spacing in the reduced model, this form is not possible as there may be no node with a run-length $l_i = l_j + 1$. We thus seek an approximation such that the prior defines an average increase in run-length of 1 if there is not a change-point. That is, we require

$$\mathbb{E}(l_i|l_j, \text{no change}) = \sum_{i=1}^{N} l_i p(l_i|l_j, \text{no change}) = l_j + 1 \tag{28}$$

For $l_{i+1} > l_i + 1$ we can match this expectation exactly by setting

$$p(l_i|l_j, \text{no change}) = \begin{cases} \frac{l_{j+1} - l_j - 1}{l_{j+1} - l_j} & \text{if } i = j \\ \frac{1}{l_{j+1} - l_j} & \text{if } i = j + 1 \\ 0 & \text{otherwise} \end{cases} \tag{29}$$

For $l_{i+1} < l_i + 1$ we approximate $p(l_i|l_j, \text{no change})$ using

$$p(l_i|l_j, \text{no change}) = \begin{cases} 0 & \text{if } i = j \\ 1 & \text{if } i = j + 1 \\ 0 & \text{otherwise} \end{cases} \tag{30}$$

In this case we do not match the expected increase in run-length. For the final node, $j = N$, it is impossible to transition to a longer run-length and so we simply have a self transition with probability 1; i.e.,

$$p(l_i|l_N, \text{no change}) = \begin{cases} 1 & \text{if } i = N \\ 0 & \text{otherwise} \end{cases} \tag{31}$$

Taken together with equation 26, equations 29, 30 and 31 define the change-point prior in the reduced model.

Like the full Bayesian model, our reduced model also has a graphical interpretation. Again each node, l_i, keeps track of two quantities: 1) the mean $\mu_t^{l_i}$, computed according to equation 24, and 2) the weight $p(l_t = l_i|x_{1:t})$. As in the full model, the weights are computed by passing messages along the edge of the graph. However, the structure of the graph is slightly different, with no increasing message being sent by node l_N and an extra 'self' message from l_i to itself. The increasing message has weight

$$\begin{array}{l} \frac{1-h}{l_{j+1} - l_j} p(l_j|x_{1:t}) \text{ for } l_{j+1} > l_j + 1 \\ (1-h)p(l_j|x_{1:t}) \text{ otherwise} \end{array} \tag{32}$$

the self message has weight

$$\frac{l_{j+1} - l_j - 1}{l_{j+1} - l_j}(1-h)p(l_j|x_{1:t}) \text{ for } l_{j+1} > l_j + 1 \qquad \text{0 otherwise} \tag{33}$$

and the change-point message has weight

$$hp(l_j|x_{1:t}) \tag{34}$$

Finally the new weight for each node is computed by summing all of the incoming messages to implement equation 25.

Results

In this section we present the results of simple simulations comparing the reduced and full models and use our model to fit human behavior on a simple prediction task with change-points.

Simulations

First we consider the simplest cases of one and two nodes with Gaussian data. These cases have particularly simple update rules, and their output is easy to understand. We then consider the more general case of many nodes to show how the reduced model retains many of the useful properties of the full model, such as keeping track of an approximate run-length distribution and being able to handle different kinds of data.

One and Two Nodes

To better understand the model it is useful to consider the special cases of one and two nodes with Gaussian data. When there is only one node, the model has only one run-length, l_1. The update for the mean of this single node is given by

$$\begin{aligned} \mu_t^{l_1} &= \mu_{t-1}^{l_1} + \frac{1}{l_1 + v_p}(U(x_t) - \mu_{t-1}^{l_1}) \\ &= \mu_{t-1}^{l_1} + \frac{1}{l_1 + v_p}(x_t - \mu_{t-1}^{l_1}) \end{aligned} \tag{35}$$

where we have used the fact that, for Gaussian data with a known variance, σ^2, we have $U(x_t) = x_t$. This update rule is, of course, equivalent to a simple Delta rule with a fixed learning rate. Because there is only one node, computing the run-length distribution is trivial, as $p(l_1|x_{1:t}) = 1$ for all t and thus the predictions of this model are simply the mean of the single Delta rule.

In the two-node case the model has two nodes with run-lengths l_1 and l_2. The means of these nodes update according to independent Delta rules

$$\begin{aligned} \mu_t^{l_1} &= \mu_{t-1}^{l_1} + \frac{1}{l_1 + v_p}(U(x_t) - \mu_{t-1}^{l_1}) \\ \mu_t^{l_2} &= \mu_{t-1}^{l_2} + \frac{1}{l_2 + v_p}(U(x_t) - \mu_{t-1}^{l_2}) \end{aligned} \tag{36}$$

The prediction of the two-node model is given as the weighted sum of these two nodes

$$\mu_t = p(l_1|x_{1:t})\mu_t^{l_1} + p(l_2|x_{1:t})\mu_t^{l_2} \tag{37}$$

where the weights, $p(l_1|x_{1:t})$ and $p(l_2|x_{1:t})$, are the components of the run-length distribution that update according to equation 25. For node 1, when $l_2 \geq l_1 + 1$, $p(l_1|x_{1:t})$ updates as

$$\begin{aligned} p(l_1|x_{1:t}) &\propto p(x_t|l_1)\left(p(l_1|l_1)p(l_1|x_{1:t-1}) + p(l_1|l_2)p(l_2|x_{1:t-1})\right) \\ &= p(x_t|l_1)\left(\left(h + \frac{(1-h)(l_2 - l_1 - 1)}{l_2 - l_1}\right)p(l_1|x_{1:t-1}) + hp(l_2|x_{1:t-1})\right) \\ &= p(x_t|l_1)\left(h + \frac{(1-h)(l_2 - l_1 - 1)}{l_2 - l_1}p(l_1|x_{1:t-1})\right) \end{aligned} \tag{38}$$

where we have used the fact that $p(l_1|x_{1:t-1}) + p(l_2|x_{1:t-1}) = 1$ because the run-length distribution is normalized. For node 2, $p(l_2|x_{1:t})$ updates as

$$\begin{aligned} p(l_2|x_{1:t}) &\propto p(x_t|l_2)\left(p(l_2|l_1)p(l_1|x_{1:t-1}) + p(l_2|l_2)p(l_2|x_{1:t-1})\right) \\ &= p(x_t|l_2)\left(\frac{1-h}{l_2 - l_1}p(l_1|x_{1:t-1}) + (1-h)p(l_2|x_{1:t-1})\right) \\ &= p(x_t|l_2)(1-h)\left(1 - \frac{l_2 - l_1 - 1}{l_2 - l_1}p(l_1|x_{1:t-1})\right) \end{aligned} \tag{39}$$

Thus, for the two-node case, the run-length distribution is closely tied to the likelihood of the data for each of the nodes, $p(x_t|l_1)$ and $p(x_t|l_2)$. These likelihoods are computed in a straightforward manner given the mean and run-length of each node. For Gaussian data these likelihoods take the form

$$p(x_t|l_i) = \frac{1}{\sigma\sqrt{2\pi}}\sqrt{\frac{l_i + v_p}{1 + l_i + v_p}}\exp\left(-\frac{1}{2\sigma^2}\left(\frac{l_i + v_p}{1 + l_i + v_p}\right)\left(x_t - \mu_t^{l_i}\right)^2\right) \tag{40}$$

An illustration of the output of the one and two node models is shown in figure 5A. This figure shows the predictions of one- and two-node models when faced with a relatively simple change-point task. To generate this figure, the one-node model had a single run-length, $l_1 = 5$, whereas the two-node model had two run-lengths, $l_1 = 1.5$ and $l_2 = 5$.

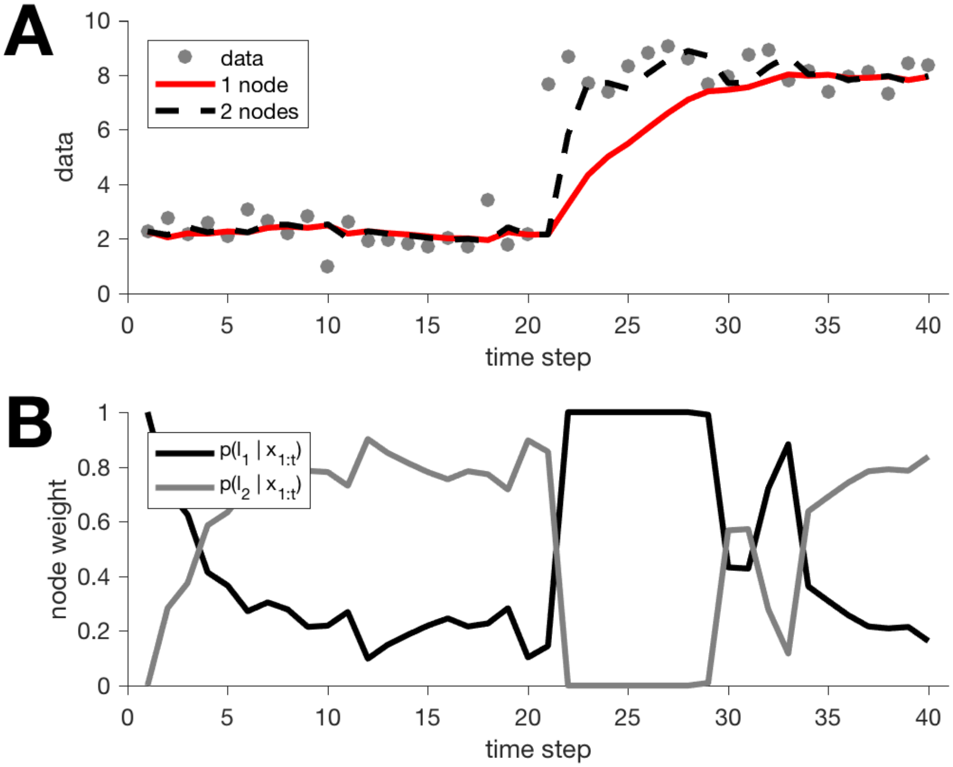

Figure 5. Output of one- and two- node models on a simple change-point task. (A) Predictions from the one- and two-node models. (B) Evolution of the node weights for the two-node model.

The hazard rate in each model was set to 0.1, and the noise standard deviation, σ, was set at 0.5. The two-node model is much better able to adapt to the change-point than the one-node model. Figure 5B shows the evolving weights of the two nodes, determined from the run-length distribution. Before the change-point, the model has a high weight on the l_2 node and a low weight on the l_1 node. At the change-point, this trend reverses abruptly but then returns after the model stabilizes to the mean of the new data.

Many nodes

Here we illustrate the utility of the approximate algorithm to solve simulated change-point problems using three different types of generative distribution. The first is a Bernoulli process with a piecewise constant rate, μ, (Figure 6A) in which the generative distribution takes the following exponential family form

$$\eta = \log\left(\frac{\mu}{1-\mu}\right); \quad H(x) = 1; \quad U(x) = x; \quad A(\eta) = -\log(1-\mu) = \log(1+\exp\eta) \quad (41)$$

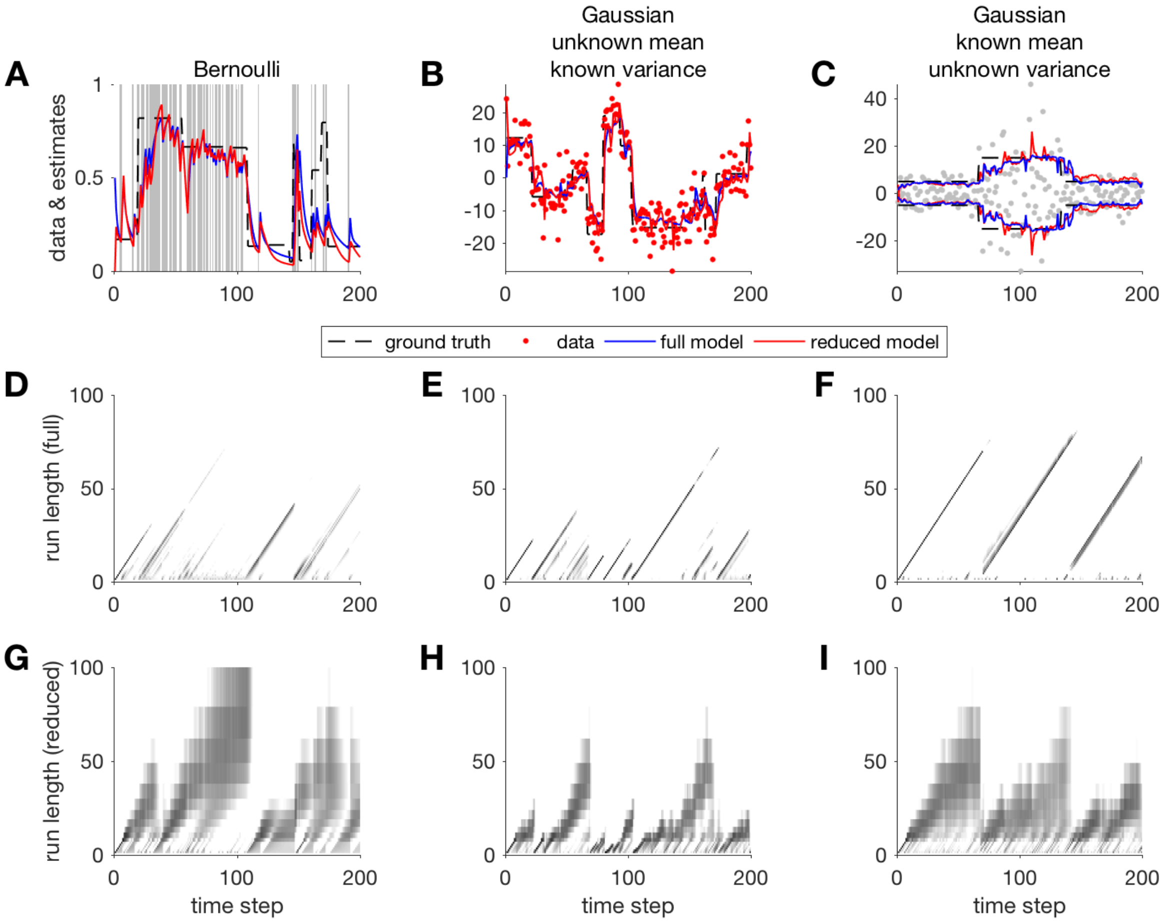

Figure 6. Examples comparing estimates and run-length distributions from the full Bayesian model and our reduced approximation for the cases of Bernoulli data (A, D, G), Gaussian data with unknown mean (B, E, H), and Gaussian data with a constant mean but unknown variance (C, F, I). (A, B, C) input data (grey), model estimates (blue: full model; red: reduced model), and the ground truth generative parameter (mean for A and B, standard deviation in C; dashed black line). Run-length distributions computed for the full model (D, E, F) and reduced model (G, H, I) are shown for each of the examples.

and with a uniform prior distribution defined by $v_p = 2$ and $\chi_p = 1$.

The second is a Gaussian distribution with known standard deviation, $\sigma = 5$, but unknown mean (Figure 6B). In this case, the generative distribution takes on the following exponential family form

$$\eta = \frac{\mu}{\sigma^2}; \quad H(x) = \frac{1}{\sqrt{2\pi}} \exp\left(-\frac{x^2}{2\sigma^2}\right); \quad U(x) = x; \quad A(\eta) = \frac{\mu^2}{2\sigma^2} \tag{42}$$

with prior hyperparameters $v_p = 1$ and $\chi_p = 0$.

The third is a Gaussian distribution with a known mean, $\mu = 0$, and a changing standard deviation σ (Figure 6C). In this case, the generative distribution takes on the following exponential family form

$$\eta = \frac{1}{\sigma^2}; \quad H(x) = \frac{1}{\sqrt{2\pi}}; \quad U(x) = -\frac{(x-\mu)^2}{2}; \quad A(\sigma) = \log \sigma \tag{43}$$

with prior hyper parameters $v_p = 1$ and $\chi_p = -1$.

For all three cases, both the full and reduced models used a fixed hazard rate (equal to 0.05 for the first and third cases, 0.025 for the second case). The reduced models used as initial sufficient statistics $\chi^{l_i} = l_i/2$ for case 1 and 2 and $\chi^{l_i} = -l_i/2$ in case 3, and had 18 nodes spaced logarithmically between 1 and 100.

In figure 6, the top row shows the true value of the parameter of interest for the generative process (the Bernoulli rate in panel A, the mean in panel B, and the standard deviation in panel C), the generated data, and the inferred value of the parameter from the full (blue) and reduced (red) models. For all three cases, there is a close correspondence between the values inferred by the full and reduced models. For the Bernoulli case, the full model has an average mean squared error (relative to ground truth) of 0.034 versus 0.036 for the reduced model. For the Gaussian case with known variance the mean squared errors are 29 for the full model and 32 for the reduced model. For the Gaussian case with known variance the errors are 7.98 and 8.83 respectively. We also show the run-length distributions inferred by both models (middle and bottom rows), which are more sparsely sampled by the reduced models but still pick up the major trends seen in the full model.

Performance of the reduced model relative to ground truth

Here we investigate the performance of the model by considering the average discrepancy between the predictions made by the reduced model and the ground truth generative parameters. Using an analytic result for the one-node case and extensive simulations for two- and three-node cases, we compute approximately optimal node arrangements for different hazard rates.

General expression for error

Although there are many measures we could use to quantify the error between the approximation and the ground truth, for simplicity, we focus here on the squared error. More specifically, we compute the expected value, over data and time, of the squared error between the predictive mean of the reduced model, m_t, and the ground truth mean on the next time step, m^G_{t+1}; i.e.,

$$E^2 = \left\langle (m_t - m^G_{t+1})^2 \right\rangle \tag{44}$$

Because our model is a mixture model, the mean m_t is given by

$$m_t = \sum_{l_i} \mu_t^{l_i} p(l_i | x_{1:t-1}) \tag{45}$$

For notational convenience we drop the t subscripts and refer to node l_i simply by its subscript i, and we write $\mu_t^{l_i} = \mu_i$ and $p(l_i|x_{1:t-1}) = p_i$. We also refer to the learning rate of node i, $\alpha_i = 1/(v_p + l_i)$. Finally, we refer to the set of nodes in the reduced model as A, such that the above equation, in our new notation, becomes

$$m = \sum_{i \in A} \mu_i p_i \tag{46}$$

Substituting this expression into equation 44 for the error we get

$$E^2 = \sum_{i\in A} \sum_{j \in A} \langle p_i p_j \mu_i \mu_j \rangle - 2 \sum_{i \in A} \left\langle p_i \mu_i m^G \right\rangle + \left\langle \left(m^G\right)^2 \right\rangle \tag{47}$$

Unfortunately, deriving an analytic expression for the error is not trivial as the terms $\langle p_i p_j \mu_i \mu_j \rangle$ and $\left\langle p_i \mu_i m^G \right\rangle$ are difficult to compute. However, these terms are tractable in the one-node case as we show below. Moreover, the error can be computed numerically for any number of nodes.

To better interpret the error, we compare the error relative to the variance of the prior distribution over the mean, m_G,

$$E_0^2 = \int m_G^2 p(m_G|v_p, \chi_p) dm_G - \left(\int m_G p(m_G|v_p, \chi_p) dm_G \right)^2 \tag{48}$$

where $p(m_G|v_p, \chi_p)$ is the prior over the mean. E_0^2 is the mean squared error if the algorithm simply predicted the mean of the prior distribution at each time step. This 'relative error,' E^2/E_0^2, varies between 0 (perfect prediction) and 1, when the algorithm picks the mean of the prior distribution, and allows us to compare different data types of prior distributions.

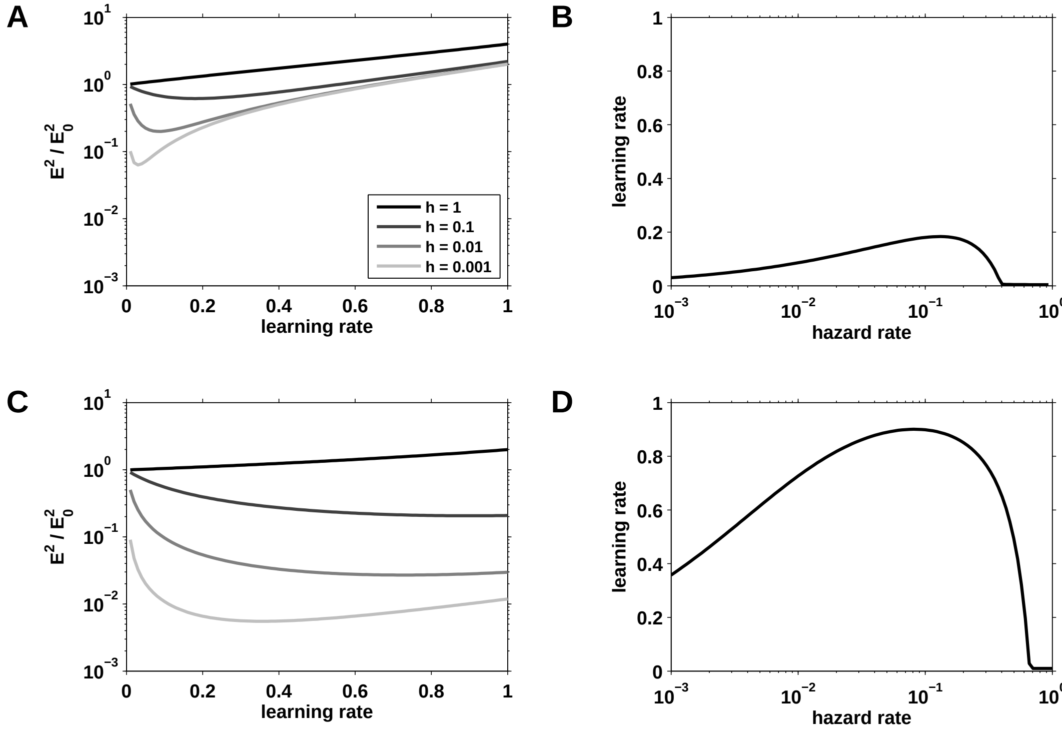

Figure 7. Error and optimal learning rates from the one-node model for Bernoulli (A, B) and Gaussian (C, D) data. (A, C) Error (normalized by the variance of the prior, E_0^2) as a function of learning rate for four different hazard rates, as indicated. (B, D) Optimal learning rate, corresponding to the lowest relative error, as a function of hazard rate.

Error for one node

We first consider how the relative error varies as a function of hazard rate and learning rate for a model with just one node (figure 7). The one-node case is useful because we can easily visualize the results and, because in this case the run-length distribution has only one non-zero term, $p_1 = 1$, it is possible to derive an exact expression for the error. In particular, for the one-node case, the expression for the error (equation 47) simplifies to

$$E(1\text{ node})^2 = \langle \mu_1^2 \rangle - 2\langle \mu_1 m^G \rangle + \left\langle \left(m^G\right)^2 \right\rangle \tag{49}$$

Thus to compute the error we only need to compute three quantities: the averages over $\langle \mu_1^2 \rangle$, $\langle \mu_1 m^G \rangle$, and $\left\langle \left(m^G\right)^2 \right\rangle$. A full derivation of these terms is presented in the Supple-

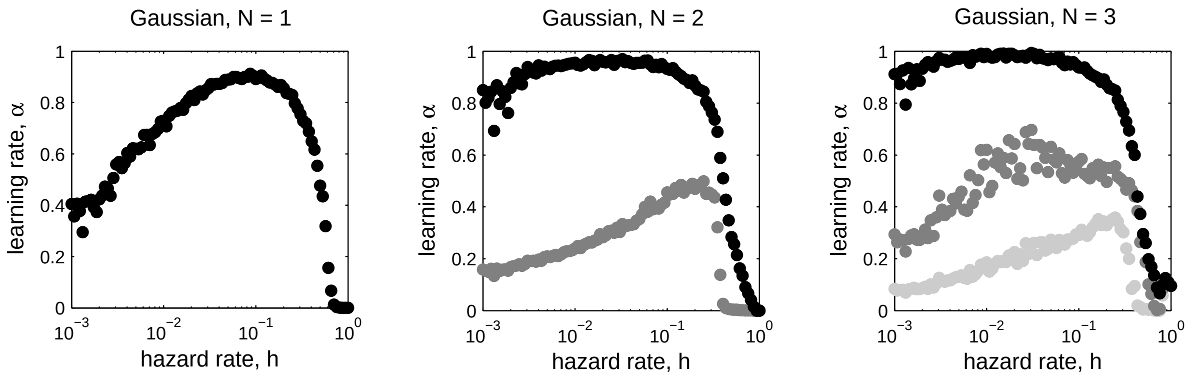

Figure 8. Optimal learning rates, corresponding to the lowest relative error (see figure 7), as a function of hazard rate and number of nodes. Gaussian case with 1 (left), 2 (center), or 3 (right) nodes.

mentary Material; here we focus on presenting how this error varies with model parameters in the specific cases of Bernoulli and Gaussian data.

Figures 7A and B consider Bernoulli data with a uniform prior ($v_p = 2$, χ_p=1). For different settings of the hazard rate, there is a unique learning rate (which is bounded between 0 and 1) that minimizes the error. The value of this optimal learning rate tends to increase as a function of increasing hazard rate, except at high hazard rates when it decreases to near zero. This decrease at high hazard rates is due to the fact that when a change happens on nearly every trial, the best guess is the mean of the prior distribution, $p(x|v_p, \chi_p)$, which is better learned with a smaller learning rate that averages over multiple change-points.

Figure 7C and D consider a Gaussian distribution with unknown mean and known variance (using parameters that match the experimental setup: standard deviation = 10, prior parameters $v_p = 0.01$ and $\chi_p = 1.5$). These plots show the same qualitative pattern as the Bernoulli case, except that the relative error is smaller and the optimal learning rate varies over a wider range. This variability results from the fact that the costs involved in making a wrong prediction can be much higher in the Gaussian case (because of the larger variance) than the Bernoulli case, in which the maximal error is between -1 and 1.

Error for multiple nodes

Next we consider the case of multiple nodes. Unlike the one-node case, deriving an analytic form for the error (equation 47) is difficult. Instead we take a numerical approach to compute approximately optimal learning rates and errors as a function of hazard rate. We minimize the relative mean squared error, computed over a finite but large enough number of time steps (much larger than the average interval between change points for each hazard rate condition). To reduce any bias effect introduced by the limited number of time steps

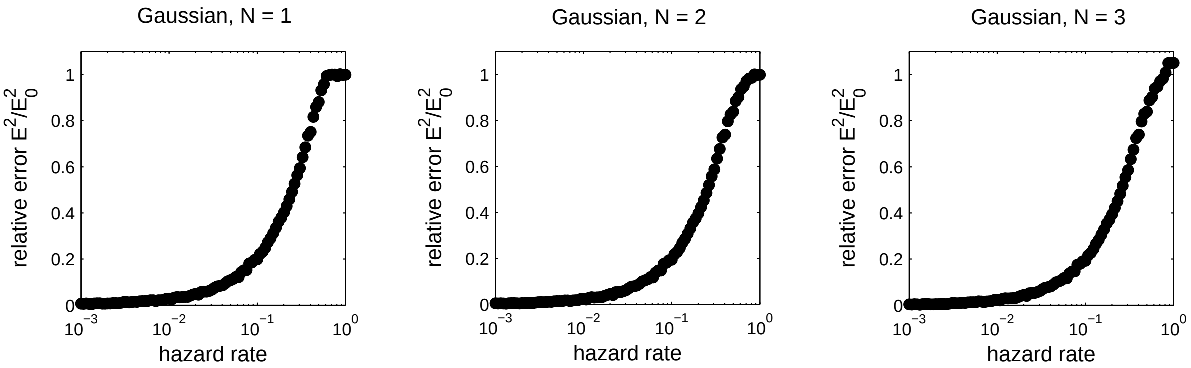

Figure 9. Error (normalized by the variance of the prior, E_0^2) computed from simulations as a function of hazard rate for the reduced model at the optimal parameter settings as shown in figure 8. Gaussian case with 1 (left), 2 (center), or 3 (right) nodes.

defining the error function, we repeat the optimization for several different realizations of the data (in each hazard rate condition) and we average the result over realizations. To minimize the relative mean squared error we use the fmincon Interior-Point Algorithm in the Matlab nonlinear optimization toolbox, with multiple re-initializations to escape local minima. To test for robustness of results we perform the same optimization with the Covariance Matrix Adaptation Evolution Strategy Algorithm, an evolutionary algorithm for numerical minimization of non-linear, non-convex (continuous domain) functions. Both algorithms return the same optimal learning rates, shown in Fig. 8, as a function of hazard rate, for the reduced model with 1-3 nodes (Gaussian data). All the learning rates show the same non-monotonic dependence on hazard rate as with one node. For three nodes, we see slightly more numerical instability (likely caused by the presence of multiple local minima) in the mid-range of hazard rates. Going to two and three nodes adds lower learning rates with a less-pronounced non-monotonic dependence on hazard rate than the single node.

In figure 9 we show the relative error as a function of hazard rate at the optimal learning rate settings computed from simulations. As in the one-node case, we see that the relative error increases with hazard rate and decreases slightly with more nodes. The biggest improvement in performance comes from increasing from one to two nodes. This suggests diminishing returns in terms of performance as more nodes are added.

Fits to experimental data

In this section, we ask how well our model describes human behavior by fitting versions of the model to behavioral data from a predictive-inference task [24]. Briefly, in this task, 30 human subjects (19 female, 11 male) were shown a sequence of numbers between 0 and 300 that were generated by a Gaussian change-point process. This process had a

mean that was randomly sampled at every change-point and a standard deviation that was constant (set to either 5 or 10) for blocks of 200 trials. Samples were constrained to be between 0 and 300 by keeping the generative means away from these bounds (the generative means were sampled from uniform distribution from 40 to 260) and resampling the small fraction of samples outside of this range until they lay within the range. The hazard rate was set at 0.1 except for the first three trials following a change-point, in which case the hazard rate was zero.

The subjects were required to predict the next number in the sequence and obtained more reward the closer their predictions were to the actual outcome. In particular, subjects were required to minimize the mean absolute error between prediction and outcome, which we denote S. Because prediction errors depended substantially on the specific sequence of numbers generated for the given session, the exact conversion between error and monetary reward was computed by comparing performance with two benchmarks: a lower benchmark (LB) and an higher benchmark (HB). The LB was computed as the mean absolute difference between sequential generated numbers. The HB was the mean difference between mean of the generative distribution on the previous trial and the generated number. Payout was then computed as follows:

$$\begin{aligned} S > LB &\implies \$8 \\ LB > S > \frac{2}{3}LB + \frac{1}{3}HB &\implies \$10 \\ \frac{2}{3}LB + \frac{1}{3}HB > S > \frac{1}{2}(LB + HB) &\implies \$12 \\ \frac{1}{2}(LB + HB) > S &\implies \$15 \end{aligned} \tag{50}$$

A benefit of this task design is that the effective learning rates used by subjects on a trial-by-trial basis can be computed in terms of their predictions following each observed outcome, using the relationships in equation 1. Our previous studies indicated that these learning rates varied systematically as a function of properties of the generative process, including its standard deviation and the occurrence of change-points [17, 24].

To better understand the computational basis for these behavioral findings, we compared five different inference models: the full Bayesian model ('full'), the reduced model with 1 to 3 nodes and the approximately Bayesian model of Nassar et al [17]. The Nassar et al model instantiates an alternative hypothesis to the mixture of fixed Delta rules by using a single Delta rule with a single, adaptive learning rate to approximate Bayesian inference.

On each trial, each of these models, M, produces a prediction m_t^M about the location of the next data point. To simulate the effects of decision noise, we assume that the subjects' reported predictions, c_t^M, are subject to noise, such that

$$c_t^M = m_t^M + \epsilon \tag{51}$$

where ϵ is sampled from a Gaussian distribution with mean 0 and standard deviation σ_d that we fit as a free parameter for all models.

In addition to this noise parameter, we fit the following free parameters for each model: The full model and the model of Nassar et al. have a hazard rate as their only other parameter, the one-node model has a single learning rate and the remaining models with N nodes ($N > 1$) have a hazard rate as well as the N learning rates.

Our fits identified the model parameters that maximized the log likelihood of the observed human predictions, c_t^H, given each of the models, $\log p(c_{1:t}^H|M)$, which is given by

$$\log p(c_{1:T}^H|M) = \sum_{t=1}^{T} \frac{(c_t^H - m_t^M)^2}{2\sigma_d^2} - T \log \sigma_d - \frac{T}{2} \log 2\pi \tag{52}$$

We used the maximum likelihood value to approximate the log Bayesian evidence, $\log E_M$ for each model using the standard Bayesian information criterion (BIC) approximation [25], which takes into account the different numbers of parameters in the different models; i.e.,

$$E_M = \frac{1}{2} BIC_M = \log(p(c_{1:T}^H|M)) - \frac{k_M}{2} \log T \tag{53}$$

where k_M is the number of free parameters in model M.

Models were then compared at the group level using the Bayesian method of Stephan et al. [26]. Briefly, this method aggregates the evidence from each of the models for each of the subjects to estimate two measures of model fit. The first, which we refer to as the 'model probability', is an estimate of how likely it is that a given model generated the data from a randomly chosen subject. The second, termed the 'exceedance probability', is the probability that one model is more likely than any of the others to have generated the behavior of all of the subjects.

An important question when interpreting the model fits is the extent to which the different models are identifiable using these analyses. In particular we are interested in the extent to which different models can be separated on the basis of their behavior and the accuracy with which the parameters of each model can be fit.

The question of model identifiability is addressed in figure 10, where we plot two confusion matrices showing the model probability (A) and the exceedance probability (B) for simulated data. These matrices were generated using simulations that matched the human-subjects experiments, with the same values of the observed stimuli, the same number of trials per experiment and the same parameter settings as found by fitting the human data. Ideally, both confusion matrices should be the identity matrix, indicating that data fit to model M is always generated by model M and never by any other model (e.g., [27]). However, because of noise in the data and the limited number of trials in the experiment, it is often the case that not all of the models are completely separable. In the present case, there is good separation for the Nassar et al., full, 1-node, and 2-node models and reasonable separation between the 3-node model and others. When

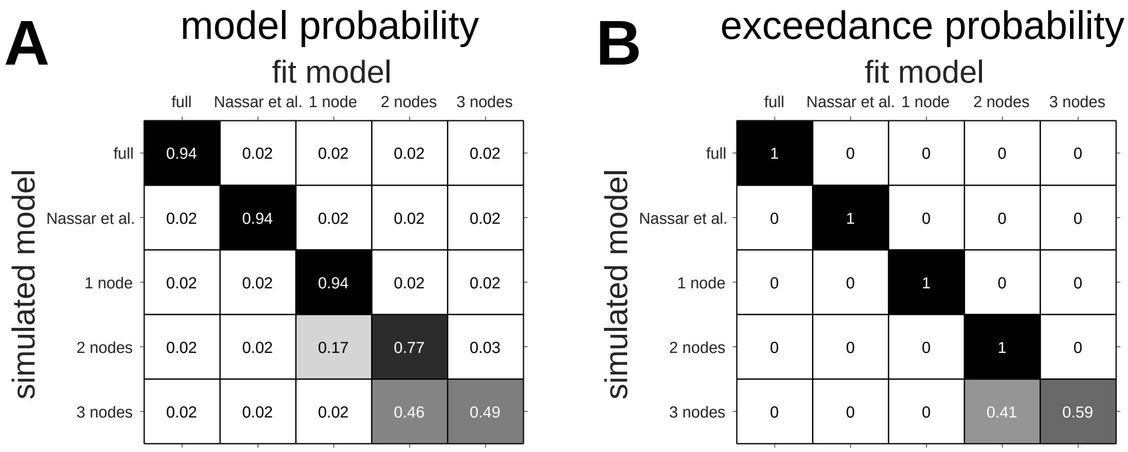

Figure 10. Confusion matrices. (A) The confusion matrix of model probability, the estimated fraction of data simulated according to one model that is fit to each of the models. (B) The confusion matrix of exceedance probability, the estimated probability at the group level that a given model has generated all the data.

we extended this analysis to include 4- and 5-node models, we found that they were indistinguishable from the 3-node model. Thus, these models are not included in our analyses, and we consider the '3-node model' to represent a model with 3 or more nodes. Note that the confusion matrix showing the exceedance probability (figure 10B) is closer to diagonal than the model probability confusion matrix (figure 10A). This result reflects the fact that exceedance probability is computed at the group level (i.e., that all the simulated data sets were generated by model M), whereas model probability computes the chance that any given simulation is best by model M.

To address the question of parameter estimability, we computed correlations between the simulated parameters and the parameter values recovered by the fitting procedure for each of the models. There was strong correspondence between the simulated and fit parameter values for all of the models and all correlations were significant (see supplementary table S1).

The 2-node model most effectively describes the human data (Figure 11), producing slightly better fits than the model of Nassar et al. at the group level. Figure 11A shows model probability, the estimated probability that any given subject is best fit by each of the models. This measure showed a slight preference for the 2-node model over the model of Nassar et al. Figure 11B shows the exceedance probability for each of the models, the probability that each of the models best fits the data at the group level. Because this measure aggregates across the group it magnifies the differences between the models and showed a clearer preference for the 2-node model. Table 1 reports the means of the corresponding fit parameters for each of the models (see also supplementary figure S1 for

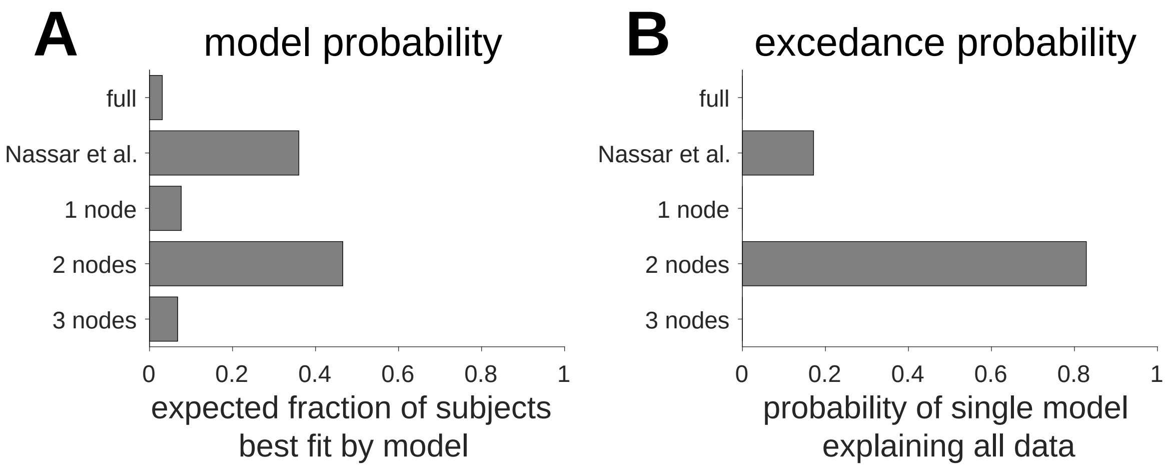

Figure 11. Results of the model-fitting procedure using the method of [28]. (A) The model probability for each of the five models. This measure reports the estimated probability that a given subject will be best fit by each of the models. (B) The exceedance probability for each of the five models. This measure reports the probability that each of the models best explains the data from all subjects.

plots of the full distributions of the fit parameters).

Model	hazard rate, h	decision noise, σ_d	learning rate(s), α
full	0.50 ± 0.04	13.39 ± 0.52	
Nassar et al.	0.45 ± 0.04	8.35 ± 0.87	
1 node		8.7 ± 0.72	0.88 ± 0.014
2 nodes	0.36 ± 0.04	7.41 ± 0.67	0.92 ± 0.01<br>0.43 ± 0.03
3 nodes	0.44 ± 0.04	7.8 ± 0.76	0.91 ± 0.01<br>0.46 ± 0.02<br>0.33 ± 0.02

Table 1. Table of mean fit parameter values for all models ± s.e.m.

Discussion

The world is an ever-changing place. Humans and animals must recognize these changes to make accurate predictions and good decisions. In this paper, we considered dynamic worlds in which periods of stability are interrupted by abrupt change-points that render

the past irrelevant for predicting the future. Previous experimental work has shown that humans modulate their behavior in the presence of such change-points in a way that is qualitatively consistent with Bayesian models of change-point detection. However, these models appear to be too computationally demanding to be implemented directly in the brain. Thus we asked two questions: 1) Is there a simple and general algorithm capable of making good predictions in the presence of change-points? And 2) Does this algorithm explain human behavior? In this section we discuss the extent to which we have answered these questions, followed by a discussion of the question that motivated this work: Is this algorithm biologically plausible? Throughout we consider the broader implications of our answers and potential avenues for future research.

Does the reduced model make good predictions?

To address this question, we derived an approximation to the Bayesian model based on a mixture of Delta rules, each implemented in a separate 'node' of a connected graph. In this reduced model, each Delta rule has its own, fixed learning rate. The overall prediction is generated by computing a weighted sum of the predictions from each node. Because only a small number of nodes are required, the model is substantially less complex than the full Bayesian model. Qualitatively, the outputs of the reduced and full Bayesian models share many features, including the ability to quickly increase the learning rate following a change-point and reduce it during periods of stability. These features were apparent for the reduced model even with a small number of (2 or 3) nodes. Thus, effective solutions to change-point problems can be achieved with minimal computational cost.

For future work, it would be interesting to consider other generative distributions, such as a Gaussian with unknown mean and variance or multidimensional data (e.g., multidimensional Gaussians) to better assess the generality of this solution. In principle, these extensions should be straightforward to deal with in the current model, which would simply require the sufficient statistic χ to be a vector instead of a scalar. Another obvious extension would be to consider generative parameters that drift over time (perhaps in addition to abrupt changes at change-points) or a hazard rate that changes as a function of run-length and/or time.

Does the reduced model explain human behavior?

To address this question, we used a model-based analysis of human behavior on a prediction task with change-points. The reduced model fit the behavioral data better than either the full Bayesian model or a single learning-rate Delta rule. Our fits also suggest that a two-node model can, in many cases, be sufficient to explain human performance on the task. However, our experiment had limited power to distinguish between the 2- and 3-node models. Thus, although the results imply that the two-node model is better than the other models we tested, we cannot rule out the possibility that humans use more that

two learning rates.

Despite this qualification, it is an intriguing idea that the brain might use just a handful of learning rates. If true, such a result would complement recent work showing that in many probabilistic-inference problems faced by humans [29] and pigeons [30], as few as just one sample from the posterior can be enough to generate good solutions.

It is also interesting to note that, for models with more than one node, the fastest learning rate was always close to one. Such a high learning rate corresponds to a Delta rule that does not integrate any information over time and simply uses the last outcome to form a prediction. This qualitative difference in the behavior of the fastest node could indicate a very different underlying process such as working memory for the last trial as is proposed in [31, 32].

One situation in which many nodes would be advantageous is the case in which the hazard rate changes as a function of run-length. In this case, only having a few run-lengths available would be problematic, because the changing hazard rate would be difficult to represent. Experiments designed to measure the effects of variable hazard rates on the ability to make predictions might therefore be able to distinguish whether multiple Delta rules are indeed present.

Is the reduced model biologically plausible?

The question of biological plausibility is always difficult to answer in computational neuroscience. This difficulty is especially true when the focus of the model is at the algorithmic level and is not directly tied to a specific neural architecture, like in this study. Nevertheless, one useful approach to help guide an answer to this question is to associate key components of the algorithm to known neurobiological mechanisms. Here we support the biological plausibility of our reduced model by showing that signatures of all the elements necessary to implement it have been observed in neural data.

In the reduced model, the update of each node uses a simple Delta rule with a fixed learning rate. The 'Delta' of such an update rule corresponds to a prediction error, correlates of which have been found throughout the brain, including notably brainstem dopaminergic neurons and their targets, and have been used extensively to model behavioral data [3–15].

More recently, several studies have also shown evidence for representations of different learning rates, as required by the model. Human subjects performing a statistical-learning task used a pair of learning rates, one fast and one slow, that were associated with BOLD activity in two different brain areas, with the hippocampus responsible for slow learning and the striatum for fast learning [33]. A related fMRI study showed different temporal integration in one network of brain areas including the amygdala versus another, more sensory network [34]. Complementary work at the neural level found a reservoir of many different learning rates in three brain regions (anterior cingulate cortex, dorsolateral prefrontal cortex, and the lateral intraparietal area) of monkeys performing a competitive

game [35]. Likewise, neural correlates of different learning rates have been identified in each of the ventral tegmental area and habenula [36]. Finally, outside of the reward system, other fMRI studies using scrambled movies have found evidence for temporal receptive fields of increasingly long time scales (equivalent to decreasingly small learning rates) up the sensory processing hierarchy [37].

Applied to our model, these results suggest that each node is implemented in a distinct, although not necessarily anatomically separated, population of neurons. For our task and the above-referenced studies, in which trials last on the order of seconds, we speculate that the mean of a node is encoded in persistent firing of neurons. Alternatively, for tasks requiring learning over longer timescales, other mechanisms such as changes in synaptic weights might play key roles in these computations.

Our model also depends on the run-length distribution, $p(l_i|x_{1:t})$. Functionally, this distribution serves as a weighting function, determining how each of the different nodes (corresponding to different run lengths) contributes to the final prediction. In this regard, the run-length distribution can be thought of as an attentional filter, similar to mechanisms of spatial or feature-based attention, evident in multiple brain regions that enhance the output of certain signals and suppress others. For longer timescales, this kind of weighting process might have analogies to certain mechanisms of perceptual decision-making that involve the readout of appropriate sensory neurons [38]. Intriguingly, these readout mechanisms are thought to be shaped by experience – governed by a Delta-rule learning process – to ultimately enhance the most reliable sensory outputs and suppress the others [39,40]. We speculate that a similar process might help select, from a reservoir of nodes with different learning rates, those that can most effectively solve a particular task.

The brain must also solve another challenge to directly implement the run-length distribution in our model. In particular, the update equation for the weights (Eq. 25) includes a constant of proportionality that serves to normalize the probability distribution. On a computer, ensuring that the run-length distribution is normalized is relatively straightforward: after the update we just divide by the sum of the node weights. In the brain, this procedure requires some kind of global divisive normalization among all areas coding different nodes. While such divisive normalization is thought to occur in the brain [41], it may be more difficult to implement over different brain regions that are far apart.

Mixture of Delta rules versus direct modulation of learning rate

An alternative account of variability in learning rates is that the brain uses a single Delta rule whose learning rate is modulated directly. This kind of model has been used previously to explain certain behavioral and imaging results in the context of change-point tasks [17,21]. A leading candidate for this role is the neuromodulator norepinephrine (NE), which is released from the locus coeruleus (LC) and has been proposed to encode the

unexpected uncertainty associated with change-points [42]. The wide-ranging projections of LC, which include most cortical and subcortical structures, and the neuromodulatory properties of NE, which adapts the gain of neural response functions [43], make this system ideally suited to deliver a global signal such as the learning rate. Control of LC could come from top-down projections from anterior cingulate cortex [16], amygdala [44], and posterior cingulate cortex [45], all of which have been proposed to encode learning rate.

Indirect evidence for this account comes from putative correlates of LC activity such as pupil dilation [24] and skin conductance response [44] that have been found to correlate with observed learning rate. However, such results are also consistent with our model if we assume that LC signals shifts in attentional focus to Delta rules with shorter learning rates, or a modified version of our model in which the learning rates of the different nodes adapt.

Our model-based analysis of behavioral data provides some evidence in favor of the present model over the fixed learning rate model of Nassar et al. However, because the experiment was not specifically designed to tease apart these two alternatives, and we did not consider every possible implementation of a variable learning rate model, the result should be treated with caution. To fully distinguish between these two accounts will require careful experimentation to determine whether the learning rate of individual neurons (using recordings from animals) or whole brain areas (using fMRI in humans) are variable or are fixed.

Acknowledgments

We thank Michael Landy for drawing attention to the issues in the original manuscript.

References

1. Bertsekas D, Tsitsiklis JN (1996) Neurodynamic Programming. Belmont, NJ: Athena Scientific.

2. Sutton RS, Barto AG (1998) Reinforcement Learning : An Introduction. Cambridge, Massachusetts: The MIT Press.

3. Rescorla RA, Wagner AR (1972) A Theory of Pavlovian conditioning: variations in the effectiveness of reinforcement and nonreinforcement. In: Black AH, Prokasy WF, editors, Classical conditioning II: current research and theory, New York: Appleton Century Crofts, chapter 3. pp. 64–99.

4. Miller RR, Barnet RC, Grahame NJ (1995) Assessment of the Rescolra-Wagner model. Psychological Bulletin 117: 363–386.

5. Schultz W, Dayan P, Montague PR (1997) A Neural Substrate of Prediction and Reward. Science 275: 1593–1599.

6. Holroyd CB, Coles MGH (2002) The Neural Basis of Human Error Processing: Reinforcement Learning, Dopamine, and the Error-Related Negativity. Psychological Review 109: 679 –709.

7. Doherty JO, Critchley H, Deichmann R, Dolan RJ (2003) Dissociating Valence of Outcome from Behavioral Control in Human Orbital and Ventral Prefrontal Cortices. The Journal of Neuroscience 23: 7931–7939.

8. Brown JW, Braver TS (2005) Learned Predictions of Error Likelihood in the Anterior Cingulate Cortex. Science 307: 1118–1121.

9. Debener S, Ullsperger M, Siegel M, Fiehler K, Cramon DYV, et al. (2005) Trial-by-Trial Coupling of Concurrent Electroencephalogram and Functional Magnetic Resonance Imaging Identifies the Dynamics of Performance Monitoring. The Journal of Neuroscience, 25: 11730 –11737.

10. Seo H, Lee D (2007) Temporal Filtering of Reward Signals in the Dorsal Anterior Cingulate Cortex during a Mixed-Strategy Game. The Journal of Neuroscience 27: 8366–8377.

11. Matsumoto M, Hikosaka O (2007) Lateral habenula as a source of negative reward signals in dopamine neurons. Nature 447: 1111–1115.

12. Matsumoto M, Matsumoto K, Abe H, Tanaka K (2007) Medial prefrontal cell activity signaling prediction errors of action values. Nature Neuroscience 10: 647–656.

13. Kennerley SW, Behrens TEJ, Wallis JD (2011) Double dissociation of value computations in orbitofrontal and anterior cingulate neurons. Nature Neuroscience 14: 1581–1589.

14. Silvetti M, Seurinck R, Verguts T (2011) Value and prediction error in medial frontal cortex: integrating the single-unit and systems levels of analysis. Frontiers in Human Neuroscience 5: 1–15.

15. Hayden BY, Pearson JM, Platt ML (2011) Neuronal basis of sequential foraging decisions in a patchy environment. Nature Neuroscience 14: 933–939.

16. Behrens TEJ, Woolrich MW, Walton ME, Rushworth MFS (2007) Learning the value of information in an uncertain world. Nature Neuroscience 10: 1214–1221.

17. Nassar MR, Wilson RC, Heasly B, Gold JI (2010) An Approximately Bayesian Delta-Rule Model Explains the Dynamics of Belief Updating in a Changing Environment. The Journal of Neuroscience 30: 12366 –12378.

18. Adams RP, Mackay DJC (2007) Bayesian Online Changepoint Detection. Technical report, Cambridge University, Cambridge.

19. Fearnhead P, Liu Z (2007) On-line inference for multiple changepoint problems. J R Statist Soc B 69: 589–605.

20. Wilson RC, Nassar MR, Gold JI (2010) Bayesian Online Learning of the Hazard Rate in Change-Point Problems. Neural Computation 2476: 2452–2476.

21. Krugel LK, Biele G, Mohr PNC, Li SC, Heekeren HR (2009) Genetic variation in dopaminergic neuromodulation influences the ability to rapidly and flexibly. PNAS 106: 17951–17956.

22. Barry JA, Hartigan D (1992) Product Partition Models for Change Point Problems. The Annals of Statistics 20: 260–279.

23. Wainwright MJ, Jordan MI (2008) Graphical Models, Exponential Families, and Variational Inference. Machine Learning 1: 1–305.

24. Nassar MR, Rumsey KM, Wilson RC, Parikh K, Heasly B, et al. (2012) Rational regulation of learning dynamics by pupil-linked arousal systems. Nature Neuroscience 15: 1040-1046.

25. Schwarz G (1978) Estimating the dimension of a model. The Annals of Statistics 6: 461–464.

26. Stephan KE, Penny WD, Daunizeau J, Moran RJ, Friston KJ (2009) Bayesian model selection for group studies. Neuroimage 46: 1004-17.

27. Steyvers M, Lee MD, Wagenmakers EJ (2009) A Bayesian analysis of human decision-making on bandit problems. Journal of Mathematical Psychology 53: 168–179.

28. Stephan KE, Penny WD, Daunizeau J, Moran RJ, Friston KJ (2009) Bayesian model selection for group studies. NeuroImage 46: 1004–1017.

29. Vul E, Goodman ND, Griffiths TL, Tenenbaum JB (2008) One and Done? Optimal Decisions From Very Few Samples. In: Proceedings of the 31st Annual Conference of the Cognitive Science Society.

30. Daw ND, Courville AC (2008) The pigeon as particle filter. In: Platt J, Koller D, Singer Y, Roweis S, editors, Advances in Neural Information Processing Systems 20, Cambridge, MA: MIT Press. pp. 369–376.

31. Collins AGE, Frank MJ (2012) How much of reinforcement learning is working memory, not reinforcement learning? a behavioral, computational, and neurogenetic analysis. Eur J Neurosci 35: 1024-35.

32. Collins A, Koechlin E (2012) Reasoning, learning, and creativity: frontal lobe function and human decision-making. PLoS Biol 10: e1001293.

33. Bornstein AM, Daw ND (2012) Dissociating hippocampal and striatal contributions to sequential prediction learning. European Journal of Neuroscience 35: 1011–1023.

34. Gläscher J, Büchel C (2005) Formal learning theory dissociates brain regions with different temporal integration. Neuron 47: 295-306.

35. Bernacchia A, Seo H, Lee D, Wang XJ (2011) A reservoir of time constants for memory traces in cortical neurons. Nature Neuroscience 14: 366–372.

36. Bromberg-Martin ES, Matsumoto M, Nakahara H, Hikosaka O (2010) Multiple Timescales of Memory in Lateral Habenula and Dopamine Neurons. Neuron 67: 499–510.

37. Hasson U, Yang E, Vallines I, Heeger DJ, Rubin N (2008) A Hierarchy of Temporal Receptive Windows in Human Cortex. Journal of Neuroscience 28: 2539 –2550.

38. Gold JI, Shadlen MN (2007) The neural basis of decision making. Annu Rev Neurosci 30: 535-74.

39. Law CT, Gold JI (2008) Neural correlates of perceptual learning in a sensory-motor, but not a sensory, cortical area. Nat Neurosci 11: 505-13.

40. Law CT, Gold JI (2009) Reinforcement learning can account for associative and perceptual learning on a visual-decision task. Nat Neurosci 12: 655-63.

41. Heeger D (1993) Modeling simple-cell direction selectivity with normalized, half-squared, linear operators. Journal of Neurophysiology 70: 1885-1898.

42. Yu AJ, Dayan P (2005) Uncertainty, Neuromodulation, and Attention. Neuron 46: 681–692.

43. Servan-Schreiber AD, Printz H, Cohen JD (1990) Reports A Network Model of Catecholamine Effects: Gain, Signal-to-Noise Ratio, and Behavior. Science 249: 892–895.

44. Li J, Schiller D, Schoenbaum G, Phelps EA, Daw ND (2011) Differential roles of human striatum and amygdala in associative learning. Nature Neuroscience 14: 1250–1252.

45. Pearson JM, Heilbronner SR, Barack DL, Hayden BY, Platt ML (2011) Posterior cingulate cortex: adapting behavior to a changing world. Trends in Cognitive Sciences 15: 143–151.
